# Resin-acid derivatives bind to multiple sites on the voltage-sensor domain of the Shaker potassium channel

**DOI:** 10.1085/jgp.202012676

**Published:** 2021-03-08

**Authors:** Malin Silverå Ejneby, Arina Gromova, Nina E. Ottosson, Stina Borg, Argel Estrada-Mondragón, Samira Yazdi, Panagiotis Apostolakis, Fredrik Elinder, Lucie Delemotte

**Affiliations:** 1 Department of Biomedical and Clinical Sciences, Linköping University, Linköping, Sweden; 2 Science for Life Laboratory, Department of Applied Physics, KTH Royal Institute of Technology, Stockholm, Sweden

## Abstract

Voltage-gated potassium (K_V_) channels can be opened by negatively charged resin acids and their derivatives. These resin acids have been proposed to attract the positively charged voltage-sensor helix (S4) toward the extracellular side of the membrane by binding to a pocket located between the lipid-facing extracellular ends of the transmembrane segments S3 and S4. By contrast to this proposed mechanism, neutralization of the top gating charge of the Shaker K_V_ channel increased resin-acid–induced opening, suggesting other mechanisms and sites of action. Here, we explore the binding of two resin-acid derivatives, Wu50 and Wu161, to the activated/open state of the Shaker K_V_ channel by a combination of in silico docking, molecular dynamics simulations, and electrophysiology of mutated channels. We identified three potential resin-acid–binding sites around S4: (1) the S3/S4 site previously suggested, in which positively charged residues introduced at the top of S4 are critical to keep the compound bound, (2) a site in the cleft between S4 and the pore domain (S4/pore site), in which a tryptophan at the top of S6 and the top gating charge of S4 keeps the compound bound, and (3) a site located on the extracellular side of the voltage-sensor domain, in a cleft formed by S1–S4 (the top-VSD site). The multiple binding sites around S4 and the anticipated helical-screw motion of the helix during activation make the effect of resin-acid derivatives on channel function intricate. The propensity of a specific resin acid to activate and open a voltage-gated channel likely depends on its exact binding dynamics and the types of interactions it can form with the protein in a state-specific manner.

## Teaser

Silverå Ejneby et al. use molecular dynamics simulations and electrophysiology to show that the voltage-gated Shaker potassium channel has multiple binding sites for resin-acid derivatives that can regulate its opening.

## Introduction

Resin acids, which are primarily found in pine resin, and their chemical derivatives promote the opening of several voltage-gated potassium (K_V_) channels (Ottosson et al., 2015, 2017, 2014; Sakamoto et al., 2017; Salari et al., 2018; Silverå Ejneby et al., 2018) and the voltage-gated and calcium-activated BK channel (Imaizumi et al., 2002; Sakamoto et al., 2006). A naturally occurring resin acid, isopimaric acid, also promotes inactivation of voltage-gated sodium and calcium channels (Salari et al., 2018). One example of a resin-acid–activated K_V_ channel is the human M-type K_V_ channel (hK_V_7.2/7.3), which regulates the excitability of nerve cells of the brain (Brown and Adams, 1980; Wang et al., 1998). Resin-acid derivatives are therefore interesting drug candidates for the treatment of hyperexcitability-related diseases such as epilepsy (Kobayashi et al., 2008; Ottosson et al., 2015; Silverå Ejneby et al., 2018; Wu et al., 2014a).

**Figure 1.**
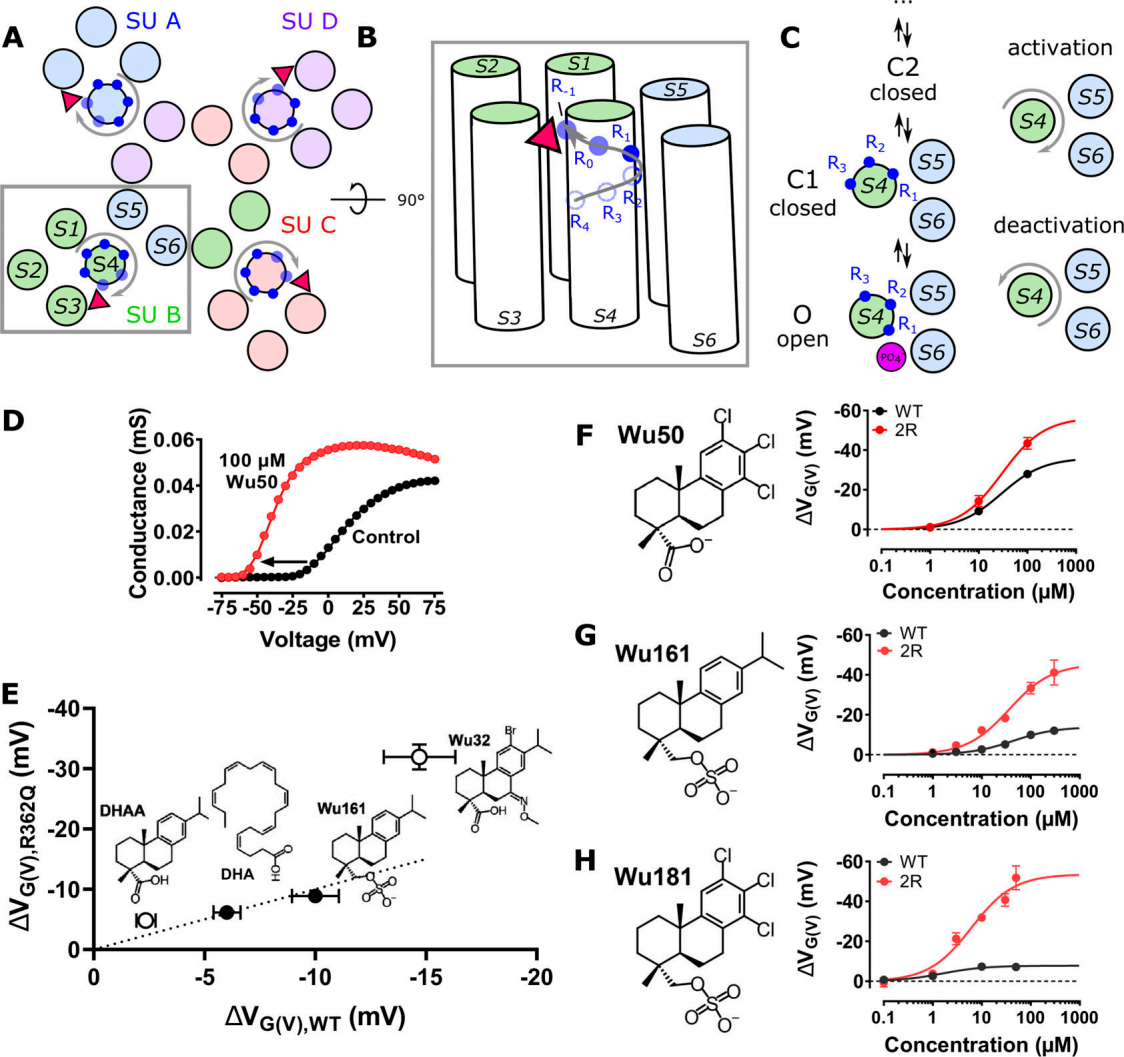
**The role of S4 mutations in the Shaker K_V_ channel for the effect of some resin-acid derivatives.** **(A and B)** Top (A) and side (B) views of the S4 helical-screw motion (gray arrows) during VSD activation. Endogenous gating charge arginines are denoted by solid or open blue circles (R362 [=R_1_], R365 [=R_2_], R368 [=R_3_], and R371 [=R_4_]). Additional charges in the 2R motif are denoted by semitransparent blue circles (M356R [=R_-1_], A359R [=R_0_]). Binding of a negatively charged compound (red triangle) in the cleft between S3 and S4 (the S3/S4 site) is hypothesized to favor S4 activation through an electrostatic effect that stabilizes the S4 activated state. The homotetrameric channel assembly is shown in A, with each subunit (SU) represented in a different color. A single subunit is shown in B (the pore domain and the VSD are from two different subunits). **(C)** Activation of Shaker channels proceeds in several activation steps between closed CX and open O states, during which the S4 helix moves in a ratchet-like upward and rotative movement (gray arrows). The purple circle marks the position of a bound lipid headgroup in the absence of compound in the open state. **(D)** 100 µM Wu50 at pH 9.0 shifted the *G*(*V*) of the Shaker K_V_ channel with the 2R motif by −40.7 mV. **(E)** *G*(*V*) shifts for R362Q versus WT Shaker K_V_ channels for four compounds. Solid symbols represent compounds for which the shift is similar in the WT and in the R362Q mutant, while empty symbols represent compounds for which the shift is larger in the mutant. The dotted line marks an equal shift in WT and R362Q. **(F–H)** Concentration-response curves (Eq. 3). Black, WT Shaker K_V_. Red, Shaker K_V_ with the 2R motif. **(F)** Wu50, pH = 9.0. WT: *EC*_50_ = 29.1 ± 3.5 µM, Δ*V*_MAX_ = −36.1 ± 1.3 mV, *n* = 3–5. 2R motif: *EC*_50_ = 30.0 ± 5.0 µM, Δ*V*_MAX_ = −56.5 ± 2.6 mV, *n* = 4–10. **(G)** Wu161, pH = 7.4. WT: *EC*_50_ = 43.7 ± 6.3 µM, Δ*V*_MAX_ = −13.9 ± 0.6 mV, *n* = 3–4. 2R motif: *EC*_50_ = 36.4 ± 9.0 µM, Δ*V*_MAX_ = −45.5 ± 3.5 mV, *n* = 3–6. **(H)** Wu181, pH = 7.4. WT: *EC*_50_ = 1.6 ± 0.6 µM, Δ*V*_MAX_ = −7.8 ± 0.5 mV, *n* = 2–3. 2R motif: *EC*_50_ = 6.1 ± 1.7 µM, Δ*V*_MAX_ = −53.6 ± 4.0 mV, *n* = 4–5. All data mean ± SEM.

In contrast to many other channel-targeting compounds, resin acids and their derivatives are relatively hydrophobic (LogP ≈ 6) because of their three-ringed structure. As such, they have been suggested to partition into the lipid bilayer and interact with the *Drosophila melanogaster* Shaker K_V_ channel from the extracellular membrane-facing side (Fig. 1, A and B, red triangles; Ottosson et al., 2017). The negatively charged resin acid is suggested to stabilize the activated state of the voltage-sensor domain (VSD) in which the positively charged S4 helix is in an up state, thereby promoting gate opening in the pore domain of the channel. Another equivalent way to describe this effect is to consider that the resin-acid derivative exerts an upward and clockwise electrostatic pull on S4 (Fig. 1 C; Ottosson et al., 2014, 2015, 2017; Silverå Ejneby et al., 2018). Thus, by stabilizing activated/open states relative to resting closed states, the resin acids shift the conductance-versus-voltage, *G*(*V*), curve toward more negative membrane voltages. In most cases, the maximum conductance, *G*_MAX_, is also increased (Fig. 1 D). The *G*(*V*) shift and the *G*_MAX_ increase can be caused by a common site and mechanism of action; however, at least for polyunsaturated fatty acids, which share some functional properties with the resin acids (Ottosson et al., 2014), it has been suggested that these two effects on the cardiac K_V_7.1 channel are mediated via two different parts of the channel (Liin et al., 2018b).

Two experimental findings support the electrostatic channel-opening mechanism. (1) The addition of two positively charged residues at the top of S4 of the Shaker K_V_ channel (M356R/A359R [=R_−1_ and R_0_ in Fig. 1 B], from hereon called the 2R motif) increases the channel-opening effect of resin acid (*G*(*V*)-shift toward more negative membrane voltages; Ottosson et al., 2014, 2015, 2017; Silverå Ejneby et al., 2018). The mechanism we propose involves an increased binding of the resin-acid derivative in the activated/open state due to the proximity of positively charged residues R_−1_ and R_0_ (Fig. 1, A and B, semitransparent circles). (2) Substituting the negative charge on the resin acid by a positive one promotes channel closing (*G*(*V*)-shift toward more positive membrane voltages; Ottosson et al., 2017), presumably through electrostatic repulsion between the positive charges of the resin acid and of the gating charges in the activated/open state of the channel. Mutagenesis and molecular modeling experiments are consistent with resin-acid derivatives binding between the extracellular portions of S3 and S4, located in the periphery of the VSD (Fig. 1, A and B; Ottosson et al., 2017).

However, some phenomena cannot easily be explained by this simple mechanism: neutralizing the outermost endogenous gating charge R362 to glutamine (R362Q = R1Q) of the Shaker K_V_ channel has surprising effects on the *G*(*V*)*-*shifting effects of some resin-acid derivatives (Ottosson et al., 2017; Silverå Ejneby et al., 2018). Using the simple model presented above, we would expect neutralization of R362 to lead to a decreased binding of the compound in the activated state (due to the loss of a positively charged residue close to the binding site) and to thus systematically decrease the *G*(*V*)-shifting effect. Instead, the neutralization of R362 led to an increase in effect for two resin acids (dehydroabietic acid [DHAA] and Wu32) and to no noticeable change for the more flexible Wu161 and for the polyunsaturated fatty acid docosahexaenoic acid (DHA; a charged lipophilic compound also interacting electrostatically with S4; Fig. 1 E; Börjesson et al., 2010; Börjesson and Elinder, 2011; Yazdi et al., 2016). Thus, a single binding site, common for all resin-acid derivatives, located at the S3–S4 interface cannot easily explain all the experimental observations.

To be able to design and develop more potent and selective compounds, it is imperative to understand the molecular action on ion channels in detail. Therefore, in this work, we sought to explore the molecular mechanism of action of resin-acid–derivative binding with respect to the following questions: (1) why do resin-acid derivatives have a larger *G*(*V*)-shifting effect on the Shaker K_V_ channel with the 2R motif compared with the WT Shaker K_V_ channel?; (2) why does the R362Q mutation increase the shift for some compounds (e.g., Wu50, Wu32, DHAA) instead of reducing it? and (3) why is the effect of compounds with a longer and flexible stalk (e.g., Wu161, DHA) less affected by the R362Q mutation than the more compact ones?

We performed docking and MD simulations to identify new possible binding sites in the Shaker K_V_ channel and characterize the molecular determinants of binding and site-directed mutagenesis in the Shaker K_V_ channel with removed fast N-type inactivation (this channel will be referred to as WT) to test these predictions. We conclude that three possible interaction sites are available around S4 of the Shaker K_V_ channel, making for a complex, state-dependent interaction pattern.

## Materials and methods

### Expression of Shaker K_V_ channels in *Xenopus laevis* oocytes

The Shaker H4 channel (GenBank accession no. NM_167595.3; Kamb et al., 1987), with removed N-type inactivation due to a Δ6-46 deletion (ShH4IR, in Bluescript II KS(+) plasmid; Hoshi et al., 1990), is referred to as the WT Shaker K_V_ channel. Addition of two positively charged arginines (M356R/A359R) at the extracellular top of the voltage sensor increases the effect of charged lipophilic compounds (Ottosson et al., 2014, 2015, 2017). Since the endogenous arginine R362 (=R1) was found to be important for large effect of polyunsaturated fatty acids on the M356R/A359R channel (Ottosson et al., 2014), this channel was referred to as the 3R Shaker K_V_ channel. However, for the resin-acid derivatives studied in the present investigation, R362 reduced the effect. Therefore, we specifically refer to the two added arginines just outside S4 (M356R/A359R) as the 2R motif. Mutations were introduced with site-directed mutagenesis and verified with sequencing as described previously (Börjesson et al., 2010). RNA injection and oocyte handling were performed as before (Börjesson et al., 2010; Ottosson et al., 2015). All animal experiments were approved by the Linköping’s local Animal Care and Use Committee.

### Electrophysiology

Electrophysiological experiments were made 1–6 d after RNA injection. K^+^ currents were measured with the two-electrode voltage-clamp technique (GeneClamp 500B amplifier; Axon Instruments) as described previously (Ottosson et al., 2015; Silverå Ejneby et al., 2018). The amplifier’s leak compensation was used, and the currents were low-pass filtered at 5 kHz. The control solution contained (in mM) 88 NaCl, 1 KCl, 15 HEPES, 0.4 CaCl_2_, and 0.8 MgCl_2_, and pH was set with NaOH. All experiments were made at room temperature (20°C–23°C). The holding potential was set to −80 mV (or −100 mV if the mutant was not fully closed at −80 mV).

### Resin-acid derivatives

Synthesis of Wu50 (Ottosson et al., 2015), Wu161, and Wu181 (Silverå Ejneby et al., 2018) was described previously. Stock solutions were stored at −20°C and diluted in control solution just before experiments. The test solution was added to the oocyte bath manually with a syringe. Wu161 and Wu181 are permanently negatively charged, while the apparent p*K*_a_ in lipid membrane for Wu50 is around 6.5 (Ottosson et al., 2015). To make sure all Wu50 molecules were negatively charged, the pH of the control solution was set to 9.0 before the experiments. This pH change had only small effects on channel kinetics and the *G*(*V*) relation. For Wu161 and Wu181, the pH was set at 7.4.

### Analysis of electrophysiological measurements

The conductance, *G*(*V*), was calculated as

$$G\left(V\right)=I/\left(V-V_{K}\right),$$ (1)

where *I* is the steady-state current measured at the end of each test-voltage sweep (100 ms after onset; Clampfit 10.5; Molecular Devices), *V* is the absolute membrane voltage, and *V*_K_ is the reversal potential for K^+^ ions (set to −80 mV). The conductance was fitted with a Boltzmann equation (GraphPad Prism 5; GraphPad Software, Inc.)

$$G_{K}\left(V\right)=G_{MAX}/\left\{1+\exp\left[\left(V_{½}-V\right)/s\right]\right\},$$ (2)

where *G*_MAX_ is the amplitude (maximal conductance) of the curve, *V* is the absolute membrane voltage, *V*_1/2_ is the midpoint, and *s* is the slope. The resin-acid–induced *G*(*V*) shift was calculated as *V*_1/2_ (compound) − *V*_1/2_ (control). The relative change in *G*_MAX_ was calculated as *G*_MAX_ (compound) / *G*_MAX_ (control). The concentration dependence for the *G*(*V*) shifts was calculated as

$$\Delta V=\;\Delta V_{MAX}/\left(1\;+EC_{50}/c\right),$$ (3)

where Δ*V* is the voltage shift, Δ*V*_MAX_ is the voltage shift at saturating concentration, *c* is the concentration of the tested compound, and *EC*_50_ is the concentration at which half-maximum response occurs.

### Molecular docking

A previously published model of the Shaker K_V_ channel in the activated/open state was used as the receptor in docking experiments (Yazdi et al., 2016). Initial coordinates for bound resin-acid derivatives Wu50 and Wu161 were obtained using molecular docking to three different binding sites in the open state localized around the outer arginine residues on S4. The docking box was placed around the center of the upper portion of the cleft between S3 and S4 for the S3/S4 site, around the geometric center of the group defined by residues R362, R365, and W454 for the S4/pore site and around the center of the upper portion of the four-helix VSD bundle for the top-VSD site. Docking was performed independently to each subunit using Autodock Vina (Trott and Olson, 2010). Since the Autodock Vina scoring function does not account for lipid bilayer environment, the ranking of the docking positions is only approximate. We thus selected docking positions based on a few physical principles: the charged headgroup of the resin-acid derivatives had to localize in the lipid headgroup region and in the direction of positively charged protein groups, while the hydrophobic body localized in the lipid tail region. The coordinates of the ligands docking pose that were subsequently used as initial conditions for the MD simulations were thus different in each subunit and are provided on the Open Science Framework repository (https://osf.io/fw8h9).

### MD simulations

The MD simulation system, mimicking the ion channel in its environment, was constructed using the CHARMM GUI Membrane Builder (Wu et al., 2014b). The open state channel and its ligand were placed in a phosphatidylcholine (POPC) bilayer, and the system was hydrated with a 150-mM KCl solution. Mutations (as described in Table 1) were also introduced using CHARMM GUI. The CHARMM36 force field was used to describe interactions between protein (Best et al., 2012), lipid (Klauda et al., 2010), and ion (Beglov and Roux, 1994) atoms, and the TIP3P model was used to describe the water molecules (Jorgensen et al., 1983). The ligand was considered in its charged form, and topology and parameters were prepared using the SwissParam software and general CHARMM force field (CGENFF; Zoete et al., 2011; Vanommeslaeghe et al., 2010). MD simulations were performed using Gromacs version 5.1.2 (Abraham et al., 2015) on the Beskow supercomputer located at the PDC computer center, Royal Institute of Technology KTH. The simulations were performed in sequential steps of minimization, equilibration, and production, keeping the default CHARMM GUI parameters (Lee et al., 2016). Initially, 500 ns of simulations were performed for each system. They were then extended for selected systems (Table 1).

**Table 1.** Systems simulated and length of trajectories

#	Site	Compound	Mutation	Length (ns)
1	S3/S4	Wu50	WT	1,000
2	S3/S4	Wu50	2R = M356R/A359R	500
3	S4/pore	Wu50	WT	500
4	S4/pore	Wu50	W454A	500
5	S4/pore	Wu50	R362Q	500
6	S4/pore	Wu50	W454A/R362Q	1,000
7	S4/pore	Wu50	R362Q/R365Q	1,000
8	S4/pore	Wu50	R362Q/R365Q/W454A	640
9	S3/S4	Wu161	WT	500
10	S4/pore	Wu161	WT	500
11	Top-VSD	Wu50	WT	500

### MD simulation analysis

Simulation data analysis was performed by scripts using the Python library MDTraj (McGibbon et al., 2015) and is available for download on OSF (https://osf.io/fw8h9). First, the shortest distance between any heavy atom of the residue in focus and any heavy atom of the ligand was calculated over time. Then, the contact frequency was extracted as the fraction of production simulation time spent with any ligand heavy atom within a 4.5-Å cutoff of any residue heavy atom. Data are reported for the four subunits independently and can be seen as four simulation replicates informing on the replicability of the results. Visualization and figure rendering were performed using Visual Molecular Dynamics (Humphrey et al., 1996).

### Modeling of contributions of compound binding to the *G*(*V*) shift

We performed least squares fit of a dataset consisting of *G*(*V*) shifts (ΔV) for eight mutants for Wu50 and five mutants for Wu161 (Table S1) to a simple additive model (Eq. 4). We also considered a reduced dataset of five mutants for Wu50 to make the comparison between the two compounds equitable (Table S1).

The *G*(*V*) shift for each mutant was expressed as a sum of contribution from the various sites:

$$\Delta V_{model}=\Delta V_{S3/S4}+\Delta V_{S4/pore}+\Delta V_{Residual},$$ (4)

where ΔV_S3/S4_ is the *G*(*V*) shift due to compound binding to the S3/S4 site, ΔV_S4/pore_ is the *G*(*V*) shift due to compound binding to the S4/pore site, and ΔV_Residual_ is the *G*(*V*) shift due to compound binding to other sites, including the top-VSD site.

Following the assumptions based on observations made using MD simulations, ΔV_S3/S4_ was set to 0 if the 2R motif was missing (i.e., in the WT channel), ΔV_S4/pore_ could adopt one of four values depending on the type of mutation (R362/W454 = WT configuration, R362Q, W454A, or R362Q/W454A), and ΔV_Residual_ was assumed to be the same for all mutants.

### Statistics

Average values are expressed as mean ± SEM. When comparing two resin-acid–induced *G*(*V*) shifts or *EC*_50_ values, a two-tailed unpaired *t* test was used.

### Online supplemental material

**Figure S1.**
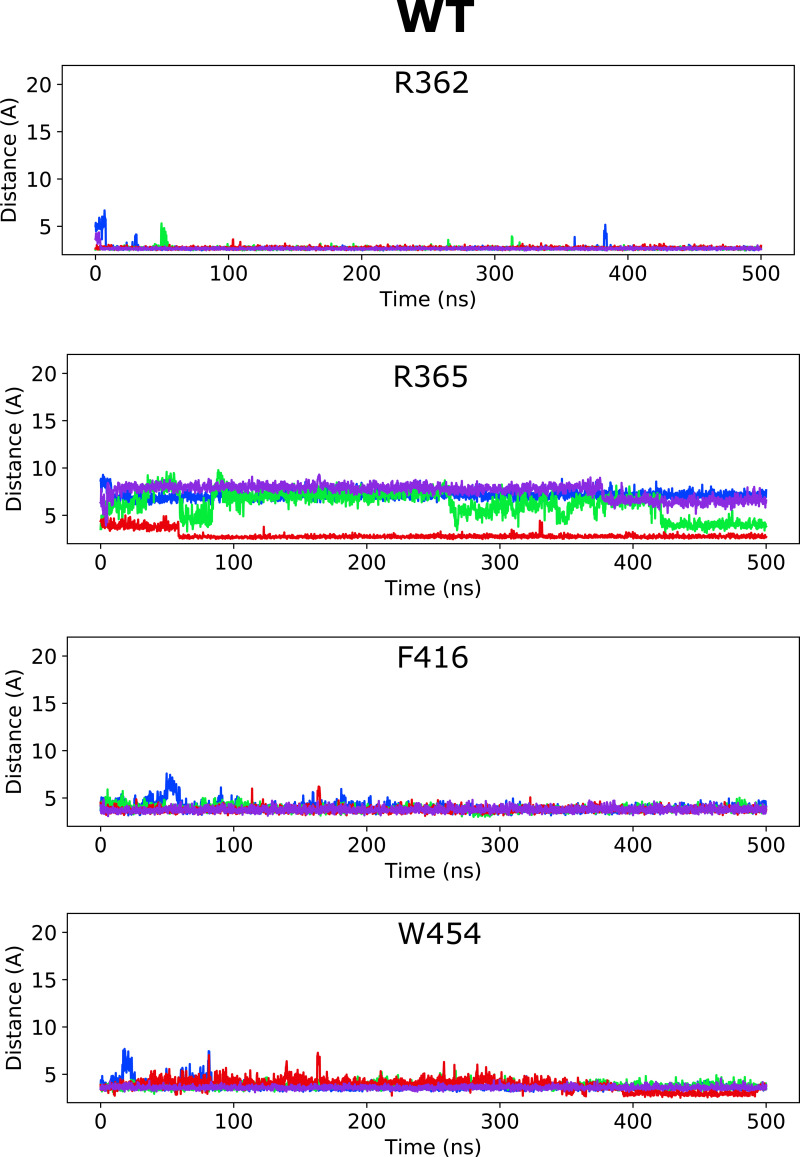
**Distance between closest atoms of Wu50 and R362, R365, F416, and W454 along the MD simulations of the WT channel system.** Each of four subunits is depicted in a different color.

**Figure S2.**
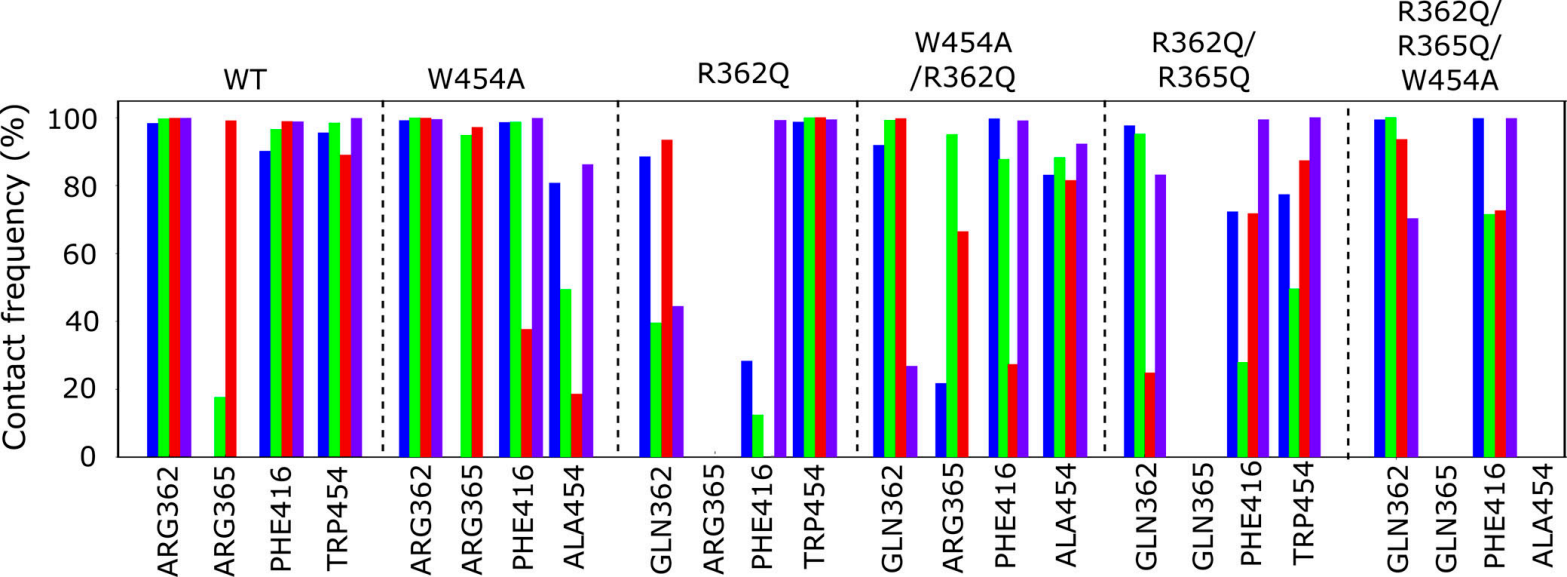
**Contact frequency between any atom of Wu50 and select S4/pore site residues in the WT, W454A, R362Q, W454A/R362Q, R362Q/R365Q, and W454A/R362Q/R365Q channel simulations.** Each of four subunits is depicted in a different color following the color scheme presented in Fig. 1.

**Figure S3.**
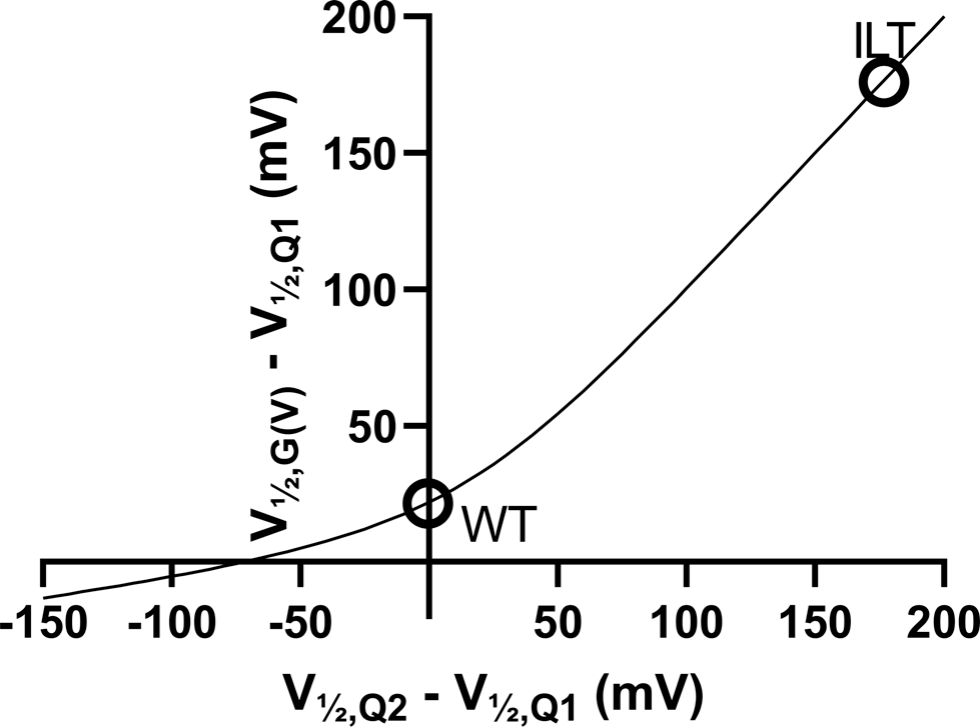
**Difference between the *G*(*V*) midpoint and the Q1 midpoint plotted against the difference between the Q2 and Q1 midpoints.** ILT denotes ILT (V369I, I372L, and S376T) mutants of the Shaker K_V_ channel.

**Figure S4.**
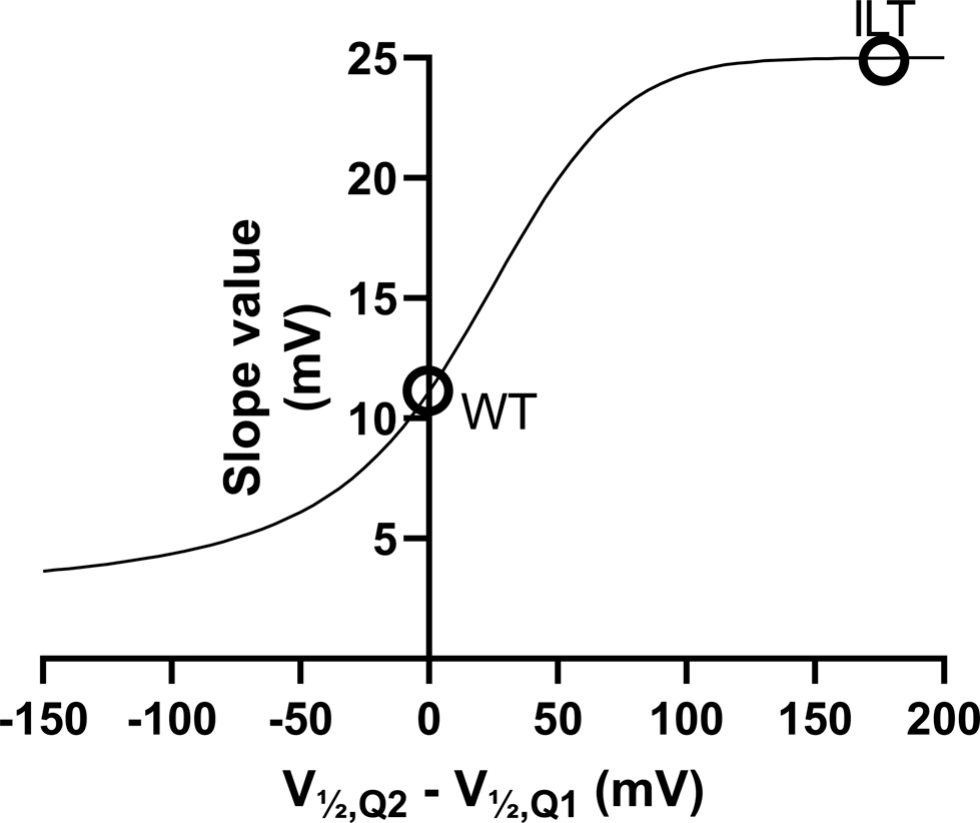
***G*(*V*) slope plotted against the difference between the Q2 and Q1 midpoints.** ILT denotes ILT (V369I, I372L, and S376T) mutants of the Shaker K_V_ channel.

**Figure S5.**
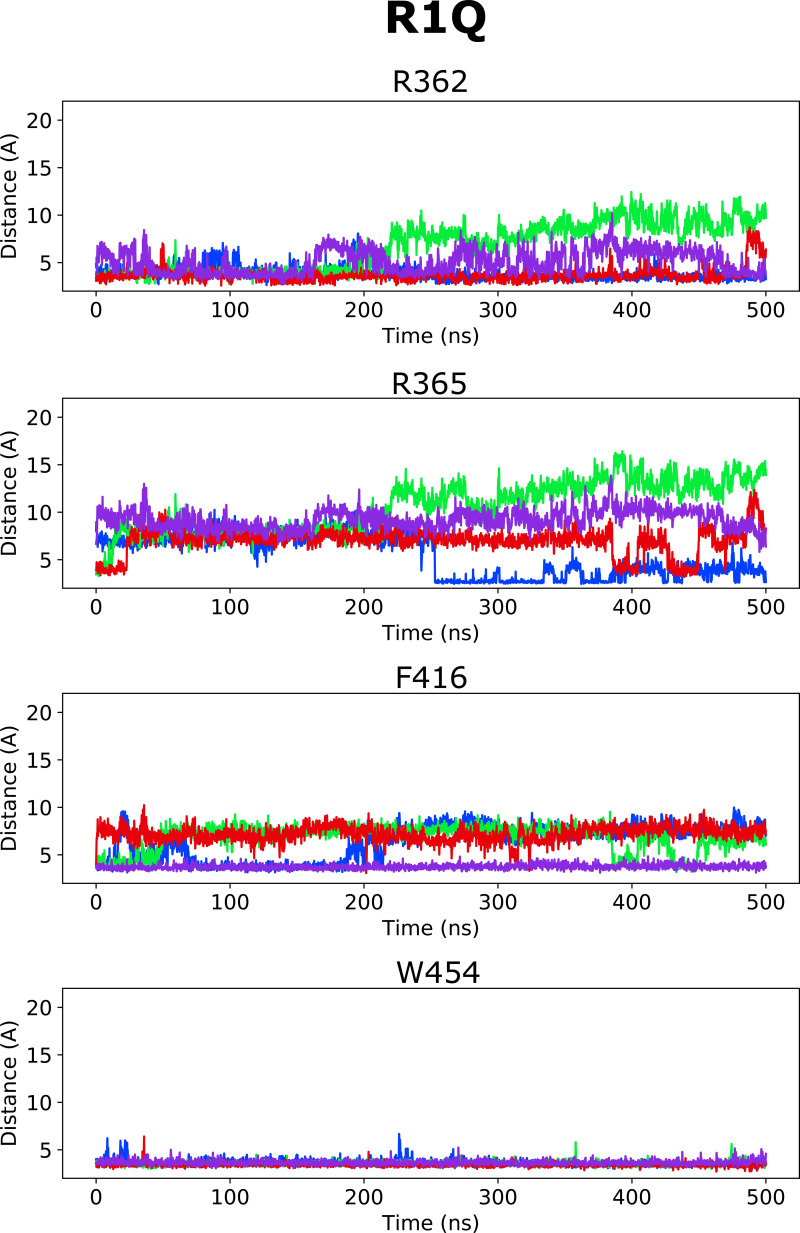
**Distance between closest atoms of Wu50 and R362, R365, F416, and W454 along the MD simulations of the R362Q channel system.** Each of four subunits is depicted in a different color.

**Figure S6.**
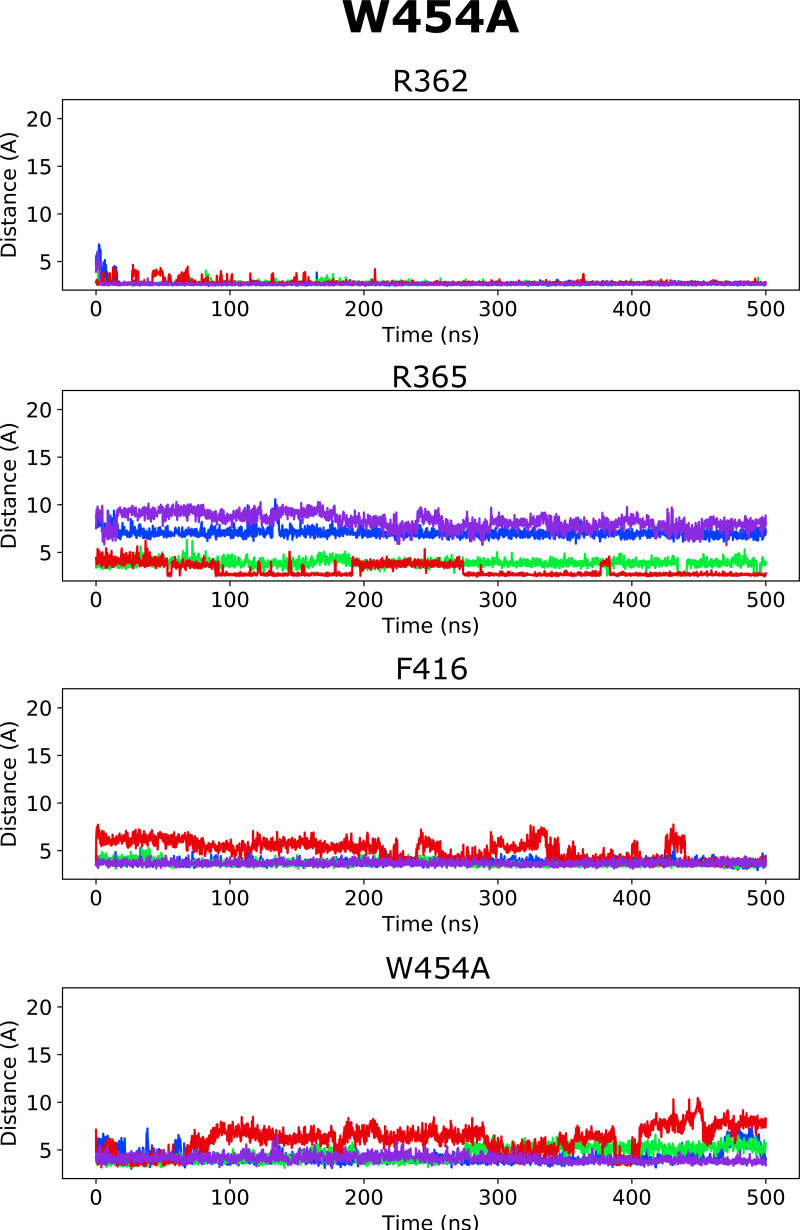
**Distance between closest atoms of Wu50 and R362, R365, F416, and W454 along the MD simulations of the W454A channel system.** Each of four subunits is depicted in a different color.

**Figure S7.**
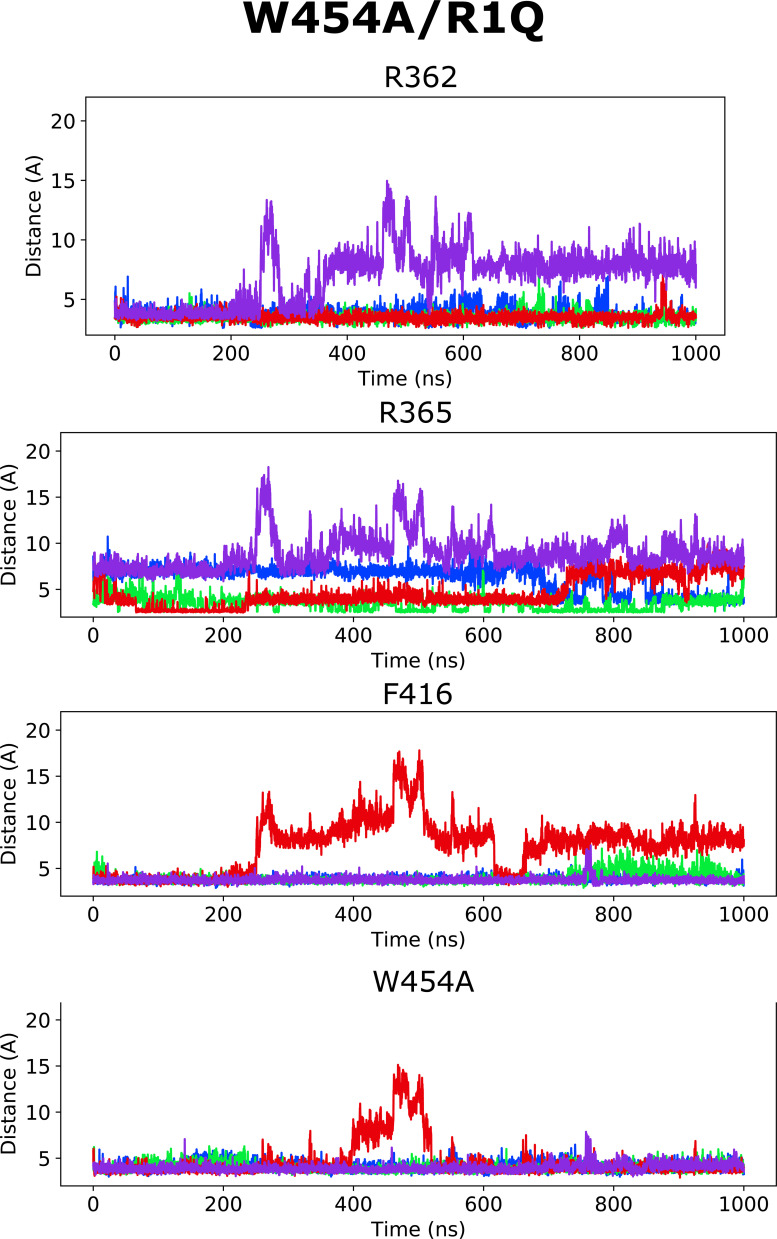
**Distance between closest atoms of Wu50 and R362, R365, F416, and W454 along the MD simulations of the W454A/R362Q channel system.** Each of four subunits is depicted in a different color.

**Figure S8.**
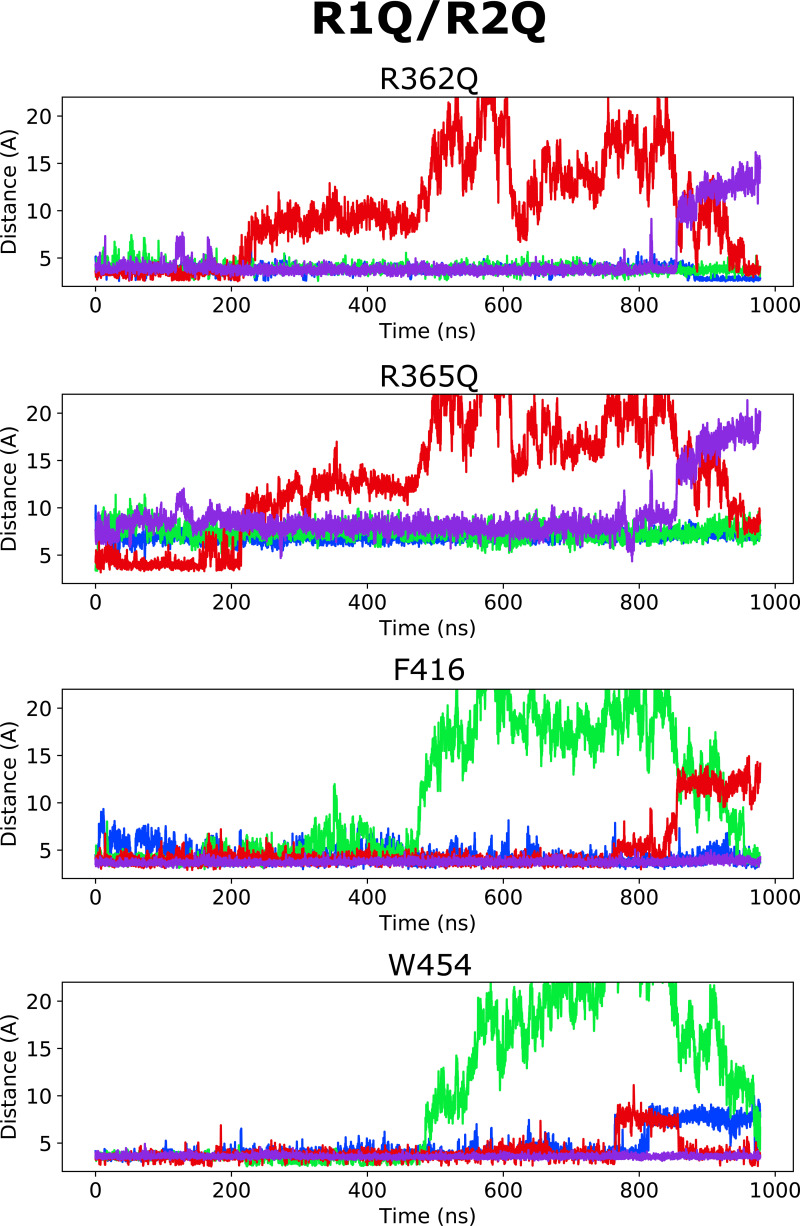
**Distance between closest atoms of Wu50 and R362, R365, F416, and W454 along the MD simulations of the R362Q/R365Q channel system.** Each of four subunits is depicted in a different color.

**Figure S9.**
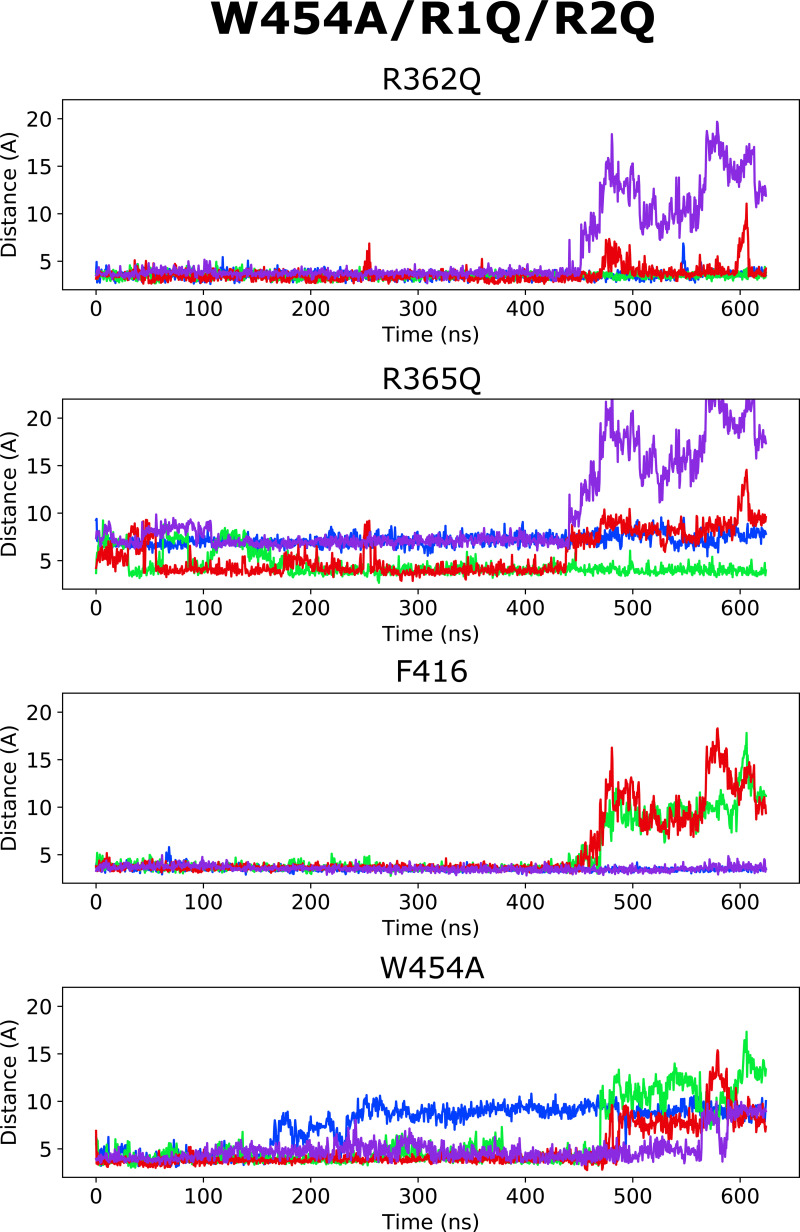
**Distance between closest atoms of Wu50 and R362, R365, F416, and W454 along the MD simulations of the R362Q/R365Q/W454A channel system.** Each of four subunits is depicted in a different color.

**Figure S10.**
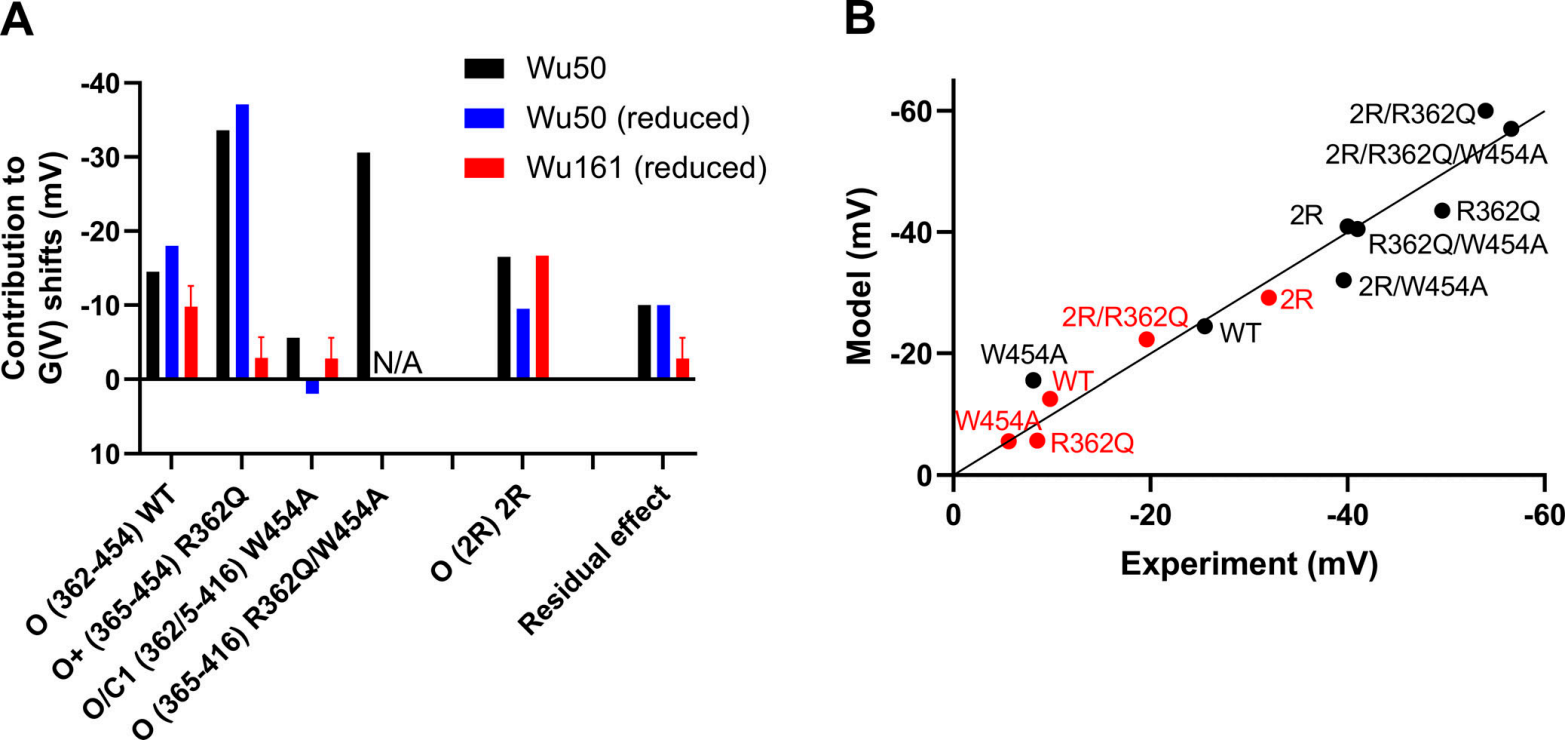
**Estimated shift contributions for separate binding sites.** **(A)** Best solutions to the models described in the Discussion of the main text. Wu50 data are based on eight mutants. Wu50 (reduced) data are based on a reduced set of five mutations (residual effect fixed to black bar). Wu161 data are based on five mutants. For O (365–416) R362Q/W454A, only the black bar exists. **(B)** Correlation between experimental data and model data as described in the Discussion of the main text. Color coding as in A.

**Figure S11.**
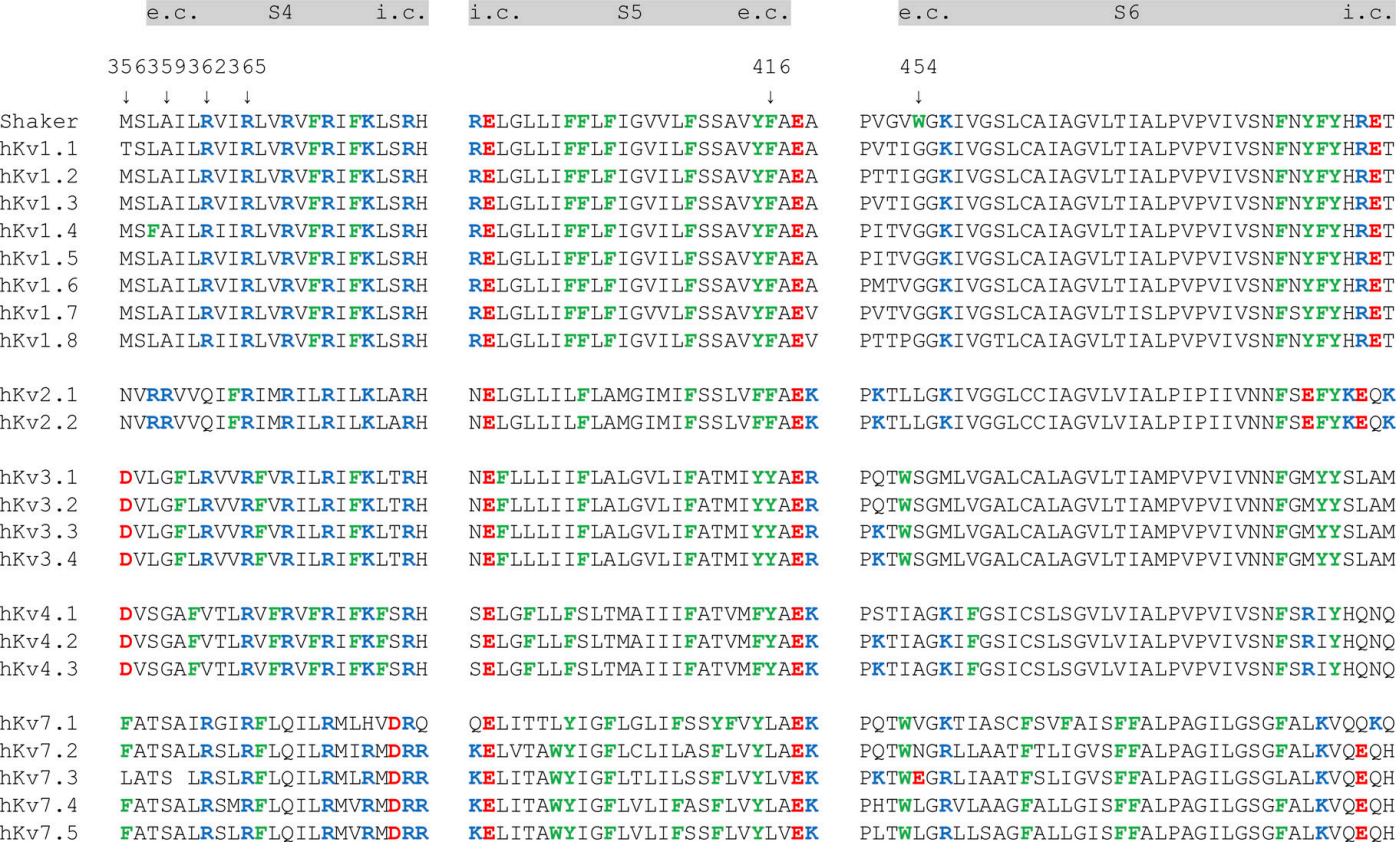
**Sequence alignment between segments S4, S5, and S6 in several K_V_ channel families, highlighting positively and negatively charged residues (blue and red, respectively) as well as aromatic ones (green).** Gray bars are approximate transmembrane helical segments. Arrows denote mutated residues in the present investigation. e.c., extracellular; i.c., intracellular.

Fig. S1, Fig. S2, Fig. S5, Fig. S6, Fig. S7, Fig. S8, and Fig. S9 report the minimum distance or contact frequency between the resin acid compound and binding site residues for the different mutants studied by MD simulations. Fig. S3 shows the difference between the *G*(*V*) midpoint and the Q1 midpoint plotted against the difference between the Q2 and Q1 midpoints. Fig. S4 shows the *G*(*V*) slope plotted against the difference between the Q2 and Q1 midpoints. Fig. S10 presents the best solutions to the models described in the Discussion. Fig. S11 reports the sequence alignment for S4, S5, and S6 for different Kv channel families. Table S1 summarizes the *G*(*V*) shift and change in *G*_MAX_ for all the mutants studied by electrophysiology. The Supplemental text lists details of the fitting procedure to obtain quantitative estimates of the contribution of binding to the different sites to the shifting effects and discusses the role of coupling between early and late voltage-sensor transitions for the interpretation of *G*(*V*) shifts.

## Results

### The 2R motif increased the maximum *G*(*V*) shift of three resin-acid derivatives

In previous work, we suggested that resin-acid derivatives open the Shaker K_V_ channel by binding to the S3/S4 cleft (Fig. 1, A and B; Ottosson et al., 2017). One of the arguments supporting this interaction site was that the double-arginine mutation M356R/A359R in the top of S4 (the 2R motif of the Shaker K_V_ channel) increased the *G*(*V*)-shifting effect of resin acids and polyunsaturated fatty acids substantially. The mechanism we proposed for this increased shift presumably involves a direct interaction of M356R and A359R with the compound in the activated/open state and thus a stabilization of this state relative to the resting/intermediate closed ones (Fig. 1, A and B; Ottosson et al., 2014). To get information on how the apparent affinity (*EC*_50_) and efficacy (maximum *G*(*V*) shift, Δ*V*_MAX_) is affected by the double-arginine mutation M356R/A359R, we explored the concentration dependence of Wu50 (Fig. 1 F) at pH 9.0 (a pH at which the compound is fully charged; Ottosson et al., 2015) and of the permanently charged compounds Wu161 (Fig. 1 G) and Wu181 (Fig. 1 H; Silverå Ejneby et al., 2018) at pH 7.4. The 2R motif increased the Wu50-induced maximum *G*(*V*) shift by 57% (from −36.1 ± 1.3 mV, *n* = 3–5, to −56.5 ± 2.6 mV, *n* = 4–10), but with no effect in apparent affinity (*EC*_50_[WT] = 29.1 ± 3.5 µM, *n* = 3–5; *EC*_50_[2R] = 30.0 ± 5.0 µM, *n* = 4–10; Fig. 1 F). In contrast to the relatively small increase in *G*(*V*) shift for Wu50, the double-arginine mutation increased the maximum *G*(*V*) shift for Wu161 by 230% (from −13.9 ± 0.6 mV, *n* = 3–4, to −45.5 ± 3.5 mV, *n* = 3–6) and for Wu181 by 590% (from −7.8 ± 0.5 mV, *n* = 2–3, to −53.6 ± 4.0 mV, *n* = 4–5). While the apparent affinity was not affected for Wu161 (*EC*_50_[WT] = 43.7 ± 6.3 µM, *n* = 3–4; *EC*_50_[2R] = 36.4 ± 9.0 µM, *n* = 3–9), it seemed to slightly decrease for Wu181, but this effect was not statistically significant (*EC*_50_[WT] = 1.6 ± 0.6 µM, *n* = 2–3; *EC*_50_[2R] = 6.1 ± 1.7 µM, *n* = 4–5).

In summary, while the maximum *G*(*V*) shift was about equal for all three compounds on the Shaker K_V_ channel with the 2R motif (−46 to −57 mV), the maximum *G*(*V*) shift on the WT Shaker K_V_ channel was much smaller for the two flexible stalk compounds (Wu161 and Wu181; −8 and −14 mV) compared with the more compact Wu50 (−36 mV). The apparent affinity, however, was not dependent on channel mutations, but compound dependent, with Wu181 being the most potent compound. Because the oocytes in many experiments did not tolerate higher concentrations of Wu181, we used Wu50 and Wu161 for the remainder of this study.

### The 2R motif increased the binding of Wu50 to the S3/S4 cleft

To gain molecular-level insights into the effect of the 2R mutation, we turned to molecular docking and MD simulations. While MD simulations do not assess compound efficacy, they provide an indication of the kinetics of unbinding from the active state of the Shaker K_V_ channel through the observation of a few stochastic events. Since the channel considered here is a homotetramer, each simulation initiated with a slightly different docking pose in each subunit provides four quasi-independent observations, and each subunit can be considered a replicate simulation. Overall, if compounds tend to detach easily from their initial binding pose, this may indicate that there is no free energy minimum at the initial docking pose location. When compounds seem to stay stably bound, this can serve as an indication of a high free-energy barrier toward unbinding, and thus indirectly of a putative high-binding affinity. We also note that docking and MD simulations are limited to the only experimentally available channel state, the open O state. Thus, the conclusions drawn are not based on explicit observations made in other states. This is unfortunate since our model relies on the relative affinity of compounds to open versus closed states. Yet, by combining insights from the computational and mutagenesis work, we are able to gradually build a mechanistic model of the effect of the resin-acid compounds on our model channel.

**Figure 2.**
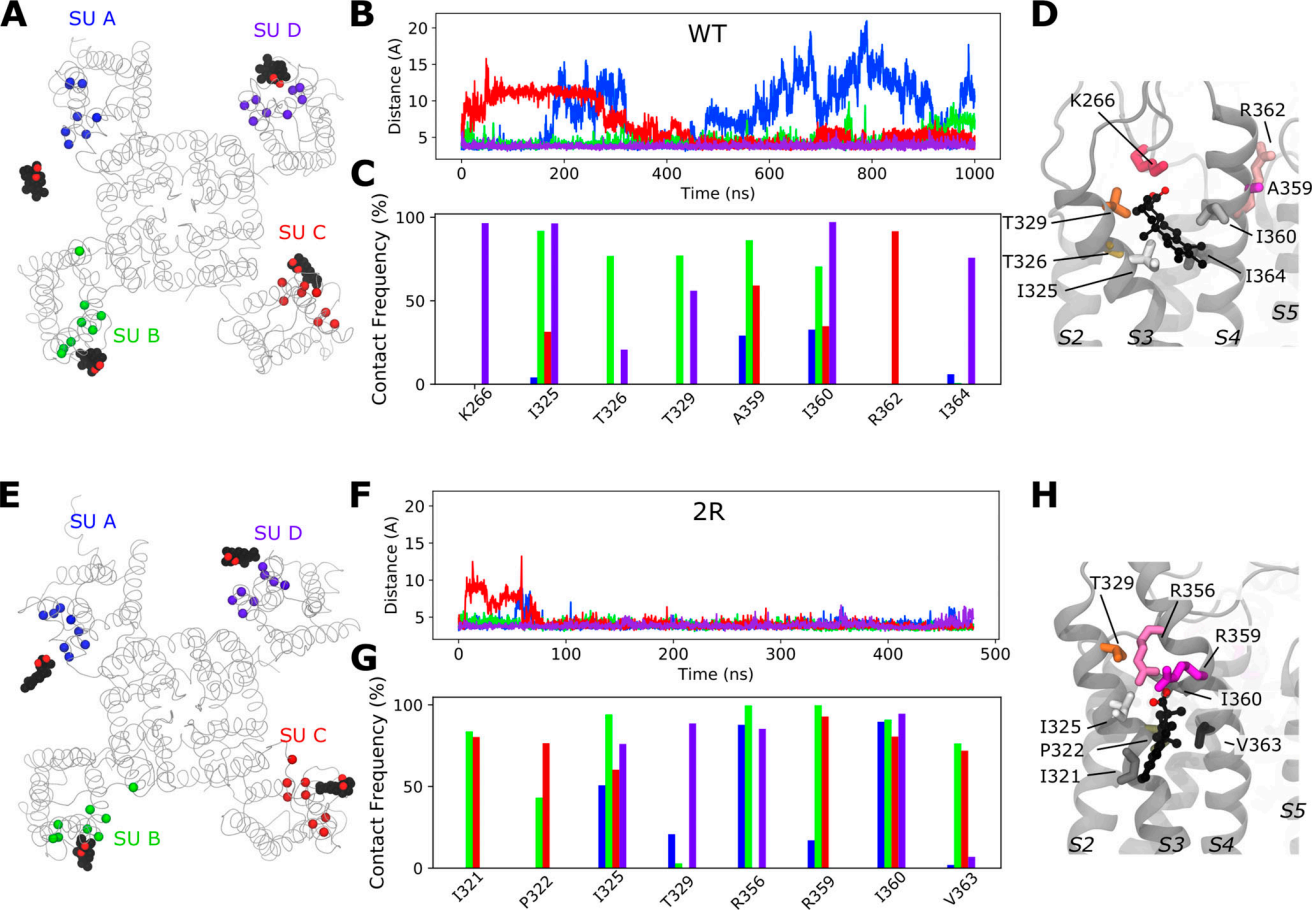
**Molecular insight into binding of Wu50 to the S3/S4 site.** **(A)** Top view of a representative snapshot of the interaction between Wu50 and the S3/S4 site in the WT Shaker K_V_ channel. The channel is shown as gray ribbons, and the Wu50 compounds are displayed using a space-filling representation, each atom type colored differently (black: C or Cl; red: O) Residues’ C_α_ coming in contact with Wu50 in at least one of the channel’s subunits (SUs) during MD simulations are represented as spheres and colored according to the subunit they belong to. **(B)** Distance between closest heavy atoms of Wu50 and ILE360 along a 1-µs-long MD simulation of the WT Shaker K_V_ channel. Each of four subunits is depicted in a different color, following the color scheme presented in A. **(C)** Contact frequency between any heavy atom of Wu50 and S3/S4 site residues in the WT Shaker K_V_ channel MD simulation. Each of the four subunits is depicted in a different color, following the color scheme presented in A. For each residue, each bar corresponds to the contact frequency in one of the four subunits. **(D)** Zoomed-in side view of the S3/S4 site for the WT Shaker K_V_ channel. The residues identified in the contact frequency analysis are shown as sticks. Apolar, polar, and positively charged residues are represented as sticks and depicted in shades of gray, orange, and pink, respectively. Wu50 compounds are displayed using a CPK representation. **(E)** Top view of a representative snapshot of the interaction between Wu50 and the S3/S4 site in the Shaker channel with the 2R motif. **(F)** Distance between closest heavy atoms of Wu50 and ILE360 along a 500-ns-long MD simulation of the 2R motif–channel system. **(G)** Contact frequency between any heavy atom of Wu50 and S3/S4 site residues in the 2R motif channel simulation. **(H)** Zoomed-in side view of the S3/S4 site for the channel with the 2R motif. Colors and representations in E–H are the same as in A–D.

Docking of Wu50 to the S3/S4 site of the fully activated/open WT Shaker K_V_ channel, followed by a 1-µs-long simulation of this system, revealed a pose in which the negatively charged carboxyl group of Wu50 was localized in the headgroup region of the lipid bilayer and interacted with polar residues Thr326 or Thr329 (in the extracellular end of S3) or with the positively charged residue Lys266 (in the S1–S2 loop), while its hydrophobic body partitioned in the lipid tail region and was in contact with hydrophobic residues of the S3/S4 upper cleft Ile325, Ala359, Ile360, and Ile364 (Fig. 2, A–D). The binding pose, however, appeared quite unstable in three out of four subunits (Fig. 2 B). In one of the subunits, the orientation of the compound even changed drastically, assuming a position parallel to the membrane surface, with the negatively charged headgroup reaching out of the pocket toward R362 (subunit C; Fig. 2, A and C). In two subunits (subunits A and B; Fig. 2, A–C), the compound eventually detached from the binding site after a few hundred nanoseconds. We noted that this occurred in the two subunits where Lys266 pointed away from the binding site, while in subunit D, where Lys266 pointed toward the binding site, the binding of Wu50 remained stable over the entire length of the MD simulation. We thus conclude that binding to the S3/S4 cleft appeared relatively weak and that the charged residues present at the top of the VSD played a role in keeping Wu50 close to the channel.

To further test the role of positively charged residues on S4, we docked Wu50 to the S3/S4 cleft in the fully activated/open Shaker K_V_ channel containing the 2R motif (Fig. 2, E–H). In all subunits, the negative charge of Wu50 quickly oriented toward M356R and/or A359R (Fig. 2, G and H). This interaction maintained the hydrophobic body in contact with I360 for more than 80% of the simulation time and with I325 for more than 50% of the simulation time in all subunits (Fig. 2 G). While different subunits displayed different behaviors, this site clearly appeared more stable than in the WT Shaker K_V_ channel. The electrostatic potential at the pocket, which is heavily influenced by the presence or absence of positively charged residues, thus seems to control the affinity of the negatively charged compound for the S3/S4 binding site.

As mentioned above, we were not able to conduct explicit docking and MD simulations in the C1 closed state of the channel. However, as S4 moves down, we expect the charges to leave the binding site both in the WT and the 2R channel and the affinity for this site to be weak in this state. The difference in binding affinity for the two states is thus larger for the 2R channel than for the WT, and thus explains why the *G*(*V*)-shifting effect is larger for the mutant.

Electrophysiology data (Fig. 1 F) indicate that the apparent efficacy of Wu50 toward the Shaker K_V_ channel with or without the 2R motif differs while its affinity to both channels is similar. The apparent difference in affinity for the activated/open state inferred from the MD simulations, as well as the unexplained behavior of the R362Q mutant for some resin acids (Fig. 1 E), could be an indication of the presence of other binding sites in the channel’s periphery.

### An S4/pore pocket is important for Wu50 effects

**Figure 3.**
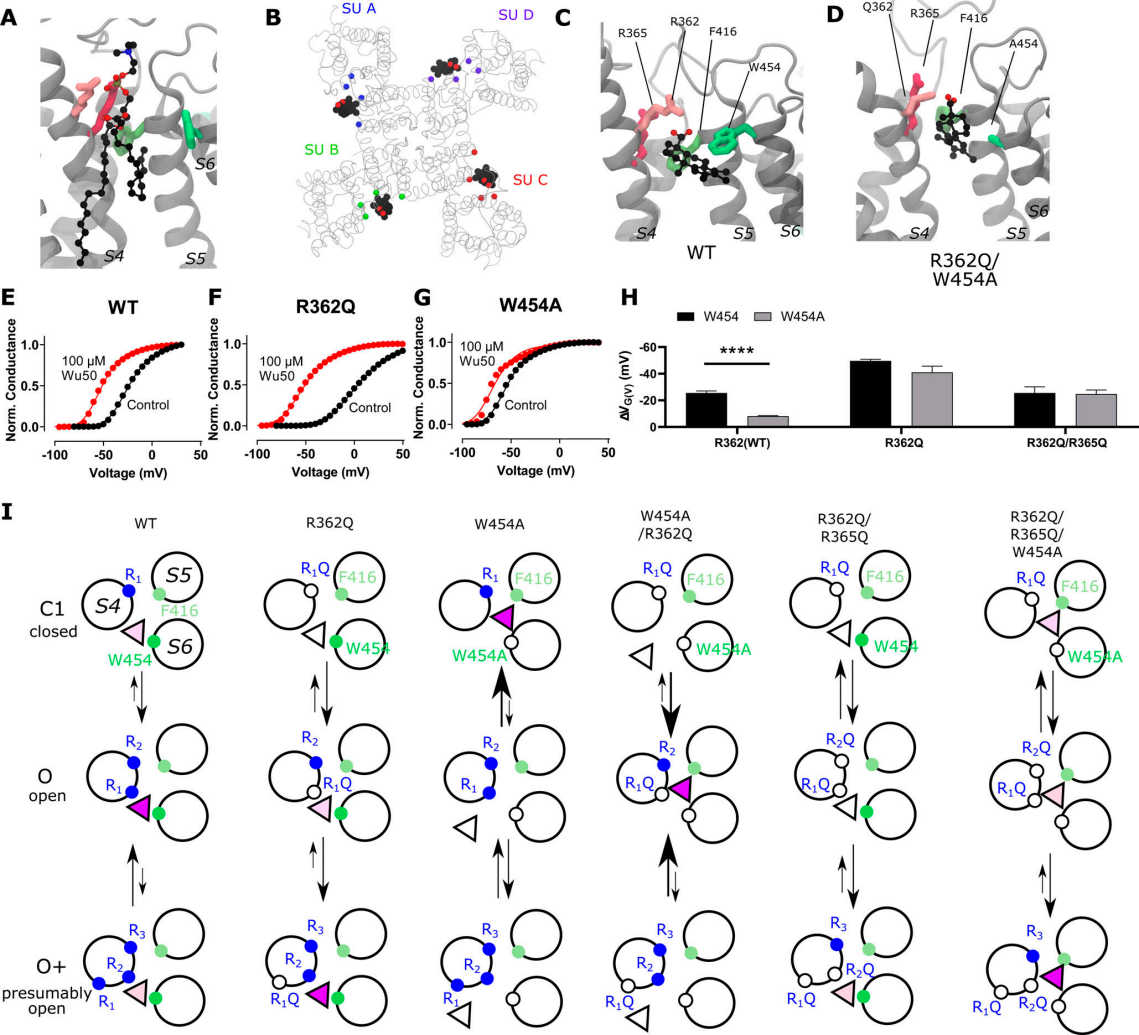
**A site located at the S4/pore interface is important for the effect of Wu50 on the WT Shaker K_V_ channel.** **(A)** Side view of the S4/pore site for the WT Shaker K_V_ channel in the absence of resin-acid derivative. A POPC molecule is occupying the binding pocket. POPC is displayed using a CPK representation, each atom type colored differently (black: C; red: O; blue: N; brown: P; hydrogens are omitted for clarity). Important interacting residues are represented as sticks (shades of green: aromatic residues, shades of pink: positively charged residues). **(B)** Top view of a representative snapshot of the interaction between Wu50 and the S4/pore site in the WT Shaker K_V_ channel. The channel is shown as gray ribbons, the Wu50 compounds are displayed using a space-filling representation, each atom type colored differently (black: C or Cl; red: O). Residues’ C_α_ coming in contact with Wu50 in at least one of the channel’s subunits (SUs) during MD simulations are represented as spheres and colored according to the subunit they belong to. **(C)** Zoomed-in side view of the S4/pore site for the WT Shaker K_V_ channel in the presence of the resin-acid derivative Wu50. Wu50 is displayed using a CPK representation, each atom type colored differently (black: C; red: O). The rest of the representation is the same as in A. **(D)** Zoomed-in side view of the deeper S4/pore site for the W454A/R362Q mutant channel in the presence of the resin-acid derivative Wu50. **(E–G)** Representative normalized *G*(*V*) curves for Wu50-induced effects on different Shaker K_V–_channel mutants. 100 µM, pH = 9. **(H)** Wu50-induced *G*(*V*) shifts for different Shaker K_V–_channel mutants. 100 µM, pH = 9. Mean ± SEM (*n* = 3–6). ****, P < 0.0001. **(I)** Scheme of putative state-dependent interactions between Wu50 in the S4/pore site in the WT and the various mutants investigated. The endogenous gating charge arginines (R362 [=R_1_], R365 [=R_2_]) are denoted by filled blue circles, F416 and W454 by filled green (light and bright, respectively) circles. Mutations are denoted by empty circles. Mutation of W454 to Ala opens a deeper binding site in the vicinity of F416. Putative binding affinities for the site range from weak (white triangles) to medium (light purple triangle) to strong (dark purple triangle). Transitions between states are represented by arrows. Relative stabilization of a state through binding of the compound to this site leads to increased transitions to this state. Stabilization by medium binding increases the transition slightly (medium-size arrow), and stabilization by strong binding increases the transition greatly (large-size arrow). A reduction in transition probability is marked by a smaller arrow. The overall *G*(*V*) shift is due to the stabilization of open states (O and O^+^) relative to closed states (C1 and other closed states not represented here).

In the active state (in the absence of a modulator compound), R362 (and R365) of the Shaker K_V_ channel interacts electrostatically with the PO_4_^−^ group of zwitterionic POPC lipids forming the membrane (Fig. 3 A; Kasimova et al., 2014). This interaction disappears when the channel deactivates, that is, as the S4 helix moves down into the intermediate closed states in a helical-screw motion (Tombola et al., 2007). Indeed, during the first steps of deactivation, R362 and R365 leave the lipid headgroups to interact with negatively charged amino acids located on helices S1–S3 (Delemotte et al., 2011; Henrion et al., 2012). It follows therefrom that when the upper leaflet contains negatively charged lipids, the activated state is stabilized through an electrostatic effect, which is reflected through a *G*(*V*) shift toward more negative membrane voltages. On the other hand, when lipid headgroups are absent (as is the case when the membrane is made of ceramide lipids, for example), the activated state is destabilized due to the lack of binding site for R362 and R365 in the activated state (Kasimova et al., 2014). The environment around the fully activated S4 helix, close to R362 and R365, thus seems to be able to accommodate compounds that are both hydrophobic and negatively charged.

We thus hypothesized that the S4/pore pocket may harbor a potential stable binding site for a negatively charged compound such as Wu50, which has a large effect on the WT Shaker K_V_ channel (Fig. 1 F) but appears to interact weakly with the activated channel’s S3/S4 cleft in the absence of the 2R motif (Fig. 2 B). To test this hypothesis, we docked Wu50 to the fully activated/open WT Shaker K_V_ channel, centering the docking box close to the PO_4_^−^ group coordinating R362. The 500-ns MD simulations initiated from these poses indicated that Wu50 tended to stay in this pocket (Fig. 3, B and C; Fig. S1; and Fig. S2). The negatively charged carboxyl group of Wu50 made a close interaction with the positively charged R362. In addition, Wu50 displayed a prominent interaction with a tryptophan residue located in the extracellular end of S6 (W454; Fig. 3 C, Fig. S1, and Fig. S2). In some subunits, the flat aromatic sidechain ring of W454 formed a tight parallel interaction with the flat trichlorinated aromatic skeleton ring of Wu50 (Fig. 3 C). A phenylalanine residue located in the extracellular end of S5 (F416) provided an interaction on the other side of the binding site (Fig. 3 C, Fig. S1, and Fig. S2). The stable and simultaneous interaction with R362 and W454 would be lost as the channel deactivates and S4 moves down while R362 rotates away from the S4/S5 cleft. We thus hypothesize that binding to this site, which we call the S4/pore site, stabilizes the VSD activated state and therefore promotes channel opening.

To experimentally test a potential binding site between R362 and W454, we mutated these residues one by one and in combination. As mentioned in the Introduction, removing the top gating charge (R362Q) did not decrease the effect but increased it (Fig. 3, F and H; and Table S1), from −25.5 ± 1.4 mV (*n* = 5) to −49.6 ± 1.2 mV (*n* = 3). In contrast, if the tryptophan 454 in S6 was mutated to an alanine, the *G*(*V*) shift was substantially reduced (Fig. 3, G and H; and Table S1), from −25.5 ± 1.4 mV (*n* = 5) to −8.1 ± 0.4 mV (*n* = 5). Simultaneously mutating W454 and R362 (R362Q/W454A) increased the effect compared with WT (Fig. 3 G and Table S1; −41.0 ± 4.4, *n* = 5).

To explain these observations, we put forward two possible mechanisms that are not mutually exclusive and may coexist: (1) The mutation per se can increase the *G*(*V*)-shifting effect of a compound without affecting the interaction between the compound and the channel. One example of this is the ILT mutation (Smith-Maxwell et al., 1998), which increases the *G*(*V*) shift induced by polyunsaturated fatty acids and resin acids through a separation of early and late voltage-dependent steps in the channel-activating sequence (Börjesson & Elinder, 2011; Ottosson et al., 2017). As described in the Supplemental text (see bottom of the PDF), however, the effects of the R362Q mutation on the separation of early and late voltage-dependent steps are mild compared with the ILT mutant, and the estimated effect of this mechanism on the *G*(*V*) shift appears rather small (Figs. S3 and S4). (2) The other explanation is that S4 can rotate farther into a hyperactivated O^+^ state, revealing a putative binding site between R365 and W454 (Fig. 3 I). This site is suggested to be revealed by the R362Q mutation and is otherwise unstable (Fig. 3 I, second column).

Altogether, according to the model mentioned previously, it is the selective binding of the compounds to the O and O^+^ state relative to the C1 and other closed states that determines the magnitude of the *G*(*V*) shift. We detail here how this model can be used to interpret the *G*(*V*) shifts in the different mutants (Fig. 3 I): in the WT Shaker K_V_ channel, binding of Wu50 is strong in the O state and weaker in the C1 and O^+^ states (first column in Fig. 3 I). Binding to C1 is presumably weak since R_1_ is located at a distance from the binding site. Binding to O^+^ is also presumably weak, but due to other reasons possibly linked to the presence of R_1_ further outward. In contrast, the R362Q mutation reduces binding to the C1 and O states (due to the loss of a positive charge in the binding site) while increasing binding to the O^+^ state (Fig. 3 I, second column; Fig. S2; and Fig. S5). This mechanism is consistent with our previous results on two other resin acids (DHAA and Wu32) showing an increased *G*(*V*) shift upon mutation of R362 (Fig. 1 D; Ottosson et al., 2017; Silverå Ejneby et al., 2018). MD simulations of the O-state W454A mutant showed an increase in instability of Wu50 binding at this site relative to WT. We interpret this as a sign of reduced binding due to the loss of interactions with W454 (Fig. 3 I, third column; Fig. S2; and Fig. S6). Additionally, MD simulations of the O-state R362Q/W454A mutant showed that Wu50 finds a deeper stable binding site between F416 (which replaced the interaction with aromatic residue W454) and R365 (Fig. 3 D; Fig. 3 I, column four; Fig. S2; and Fig. S7). To explain the increased shift in the R362Q/W454A mutant relative to WT, we need to hypothesize that this deep binding site is not occupied in the C1 and O^+^ state. By symmetry, the site is occupied in the W454A mutant in the C1 state (thanks to the proximity of R_1_) and not the O and O^+^ state, providing an explanation for the reduced *G*(*V*) shift for this mutant (Fig. 3 I, column three).

To experimentally test the idea that F416 is involved in a deep binding site, we explored R362Q/W454A/F416A. Unfortunately, Wu50 reduced *G*_MAX_ by 83%, and we could therefore not reliably measure the *G*(*V*) shift (Table S1).

To experimentally test the idea that R365 is involved in state-dependent binding, we also neutralized R365 (R362Q/R365Q). The *G*(*V*) shift was very much reduced from −49.6 ± 1.2 mV (*n* = 3; R362Q) to −25.5 ± 4.6 mV (*n* = 5; R362Q/R365Q; Fig. 3 H). Using our mechanistic model further, we propose that weak binding in the O^+^ state (due to electrostatic interaction with R3 at a distance), but not in the O and C1 states, could explain this effect (Fig. 3 I, column five). In line with this, the R362Q/R365Q/W454A mutation showed the same sensitivity to Wu50 (−24.7 ± 3.0, *n* = 6; Fig. 3 H and Table S1) as R362Q/R365Q. Since we know that the W454A mutation leads to binding to the deeper site, by symmetry, we propose that binding is weak in the C1 and O state but strong in the O^+^ state, resulting in a similar relative stabilization of states in R362Q/R365Q and R362Q/R365Q/W454A (Fig. 3 I, column six; Fig. S2; Fig. S8; and Fig. S9).

It should be noted that mutations in the S4/pore pocket also altered the effect of the compounds on *G*_MAX_ (Supplemental text and Table S1). This will be further explored in a later study.

### The 2R motif rescues the W454A effect

**Figure 4.**
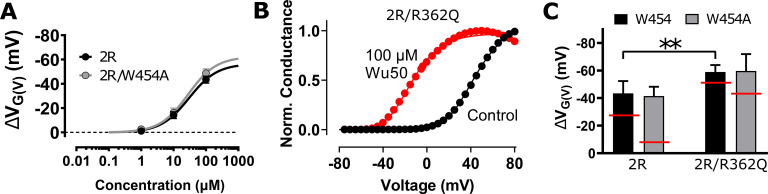
**The 2R motif overrides the effect from the S4/pore site. (A)** Concentration-response curves for Wu50 at pH 9.0. 2R motif: *EC*_50_ = 30.0 ± 5.0 µM, Δ*V*_MAX_ = −56.5 ± 2.6 mV, *n* = 4–10; 2R motif/W454A: *EC*_50_ = 27.9 ± 9.2 µM, Δ*V*_MAX_ = −62.3 ± 6.5 mV, *n* = 3. Mean ± SEM. **(B)** 100 µM Wu50 at pH 9.0 shifts *G*(*V*) for the 2R motif/R362Q Shaker KV channel results by −59.0 mV. **(C)** Wu50-induced *G*(*V*) shifts for Shaker K_V–_channel mutants as indicated. 100 µM, pH = 9. Mean ± SEM (*n* = 4–10). **, P < 0.01. The red lines denote the shifts in the absence of the double arginine mutation (M356R/A359R = 2R).

When the 2R motif was combined with the *G*(*V*) shift–attenuating mutation W454A, the attenuation was gone (Fig. 4, A and C; and Table S1). This suggests that the 2R motif trumps the effects of a compound bound to the S4/pore pocket. Combining the 2R motif with the R362Q mutation increased the *G*(*V*) shift even more (from −40.0 ± 2.7 mV [*n* = 8] to −54.0 ± 2.3 mV [*n* = 4]; Fig. 4, B and C; and Table S1), and combining the 2R motif with R362Q/W454A mutation increased the *G*(*V*) shift from −41.0 ± 4.4 (*n* = 5) to −56.6 ± 5.8 (*n* = 4; Fig. 4 C and Table S1). Thus, we hypothesize that in a channel with the 2R motif, the S3/S4 site dominates and the S4/pore pocket has no additional effect. However, in the absence of R362 (and the 2R motif) as described above, we hypothesize that the binding of the compound in the S4/pore pocket increases the *G*(*V*) shift through the recruitment of R365, which has a tendency to pull S4 into the stable O^+^ state and cause opening of the pore at lower voltages (Fig. 3, F and I). The 2R motif is therefore not per se needed for increasing the *G*(*V*)-shifting effect of resin acids.

### A longer and more flexible stalk alters Wu161 binding to the S4/pore site

Wu161 and Wu181, which have their negative charge on a three-atom-long and slightly flexible stalk, have smaller effects on the WT Shaker K_V_ channel compared with Wu50, but a very large effect when the 2R motif is introduced (Fig. 1, F–H). This suggests that the stalk compounds Wu161 and Wu181 exert their main effect on the Shaker K_V_ channel with the 2R motif via the S3/S4 site.

**Figure 5.**
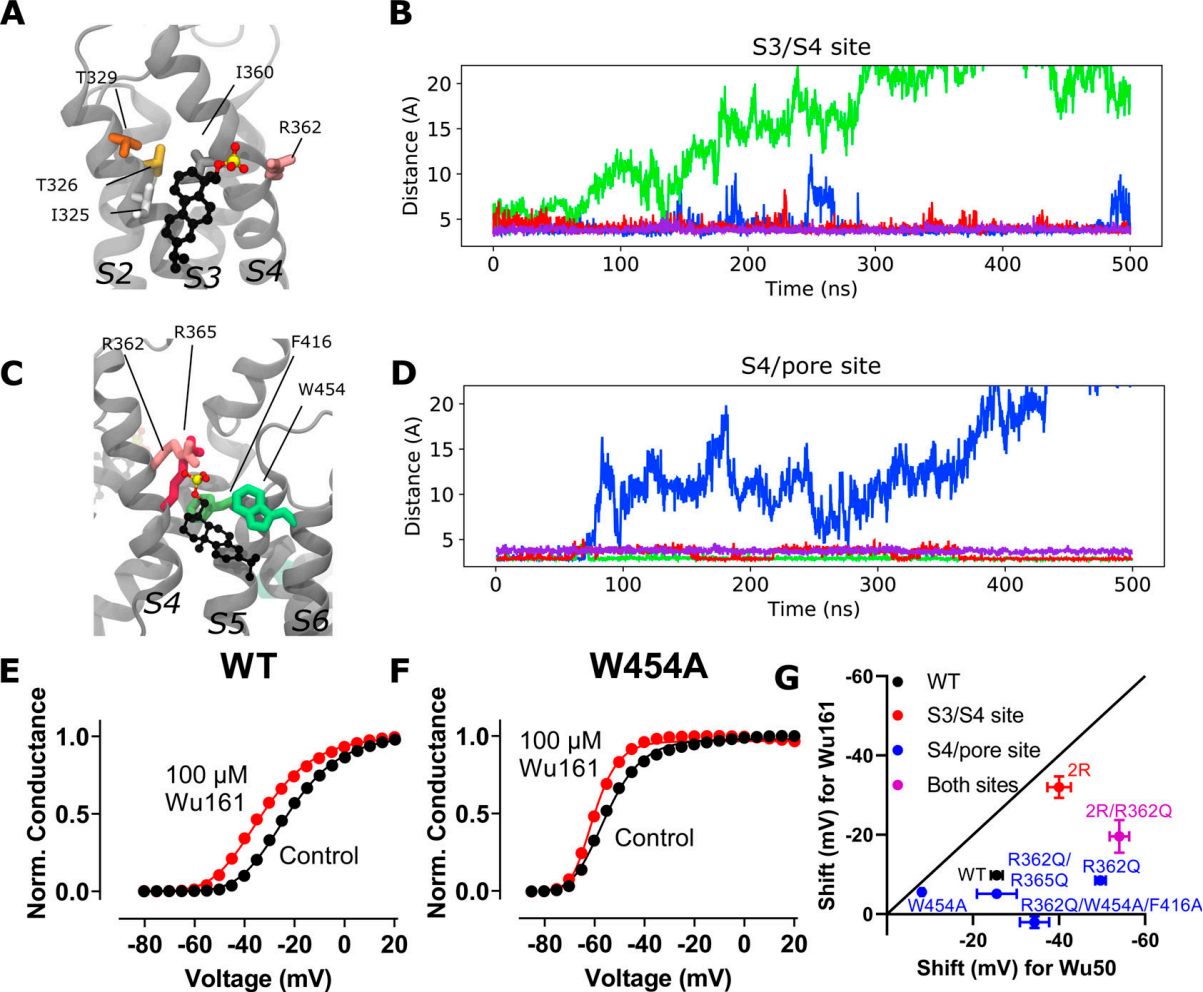
**Wu161 binding to the S3/S4 and the S4/pore sites.** **(A)** Zoomed-in side view of the S3/S4 site in the presence of Wu161. The channel is shown as gray ribbons, the Wu161 compounds are displayed in CPK representation, each atom type colored differently (yellow: S; black: C; red: O). Apolar, polar, positively charged, and aromatic residues are represented as sticks and depicted in shades of gray, orange, pink, and green, respectively. **(B)** Distance between closest heavy atoms of Wu161 and I360 along a 500-ns-long MD simulation of the WT Shaker K_V_ channel system. Each of four subunits is depicted in a different color. **(C)** Zoomed-in side view of the S4/pore site in the presence of Wu161. Representations are the same as in A. **(D)** Distance between closest heavy atoms of Wu161 and R362 along a 500-ns-long MD simulation of the WT Shaker K_V_ channel system. Each of four subunits is depicted in a different color. **(E and F)** *G*(*V*) curves before (black) and after (red) application of 100 µM Wu161 to the channels. *G*(*V*) shifts = −10.4 mV and −4.7 mV, respectively, pH = 7.4. **(G)** Correlation between *G*(*V*) shifts for Wu50 and Wu161 (100 µM) on different Shaker mutants. Error bars represent mean ± SEM (*n* = 4). The solid black line marks an equal *G*(*V*) shift for Wu50 and Wu161. Norm., normalized.

MD simulations initiated with Wu161 docked to the S3/S4 site of the fully activated/open WT Shaker K_V_ channel did not display a prominently different behavior from that of Wu50, with two out of four compounds displaying a tendency to leave their binding site (Fig. 5, A and B). On the other hand, docking of Wu161 to the S4/pore site showed substantial differences from Wu50 (Fig. 5, C and D). The binding to the WT activated/open Shaker K_V_ channel appeared overall less stable: Wu161 left the binding site in one of the subunits, and the minimum distance from the compound to R362 appeared generally larger and displayed more fluctuations than with Wu50. Indeed, scrutinizing the trajectories led to the observation that Wu161 did not fit as tightly in this binding site because its longer stalk made it impossible for their hydrophobic body to interact with W454 via pi-stacking at the same time as their negatively charged headgroup interacted with the positively charged group of R362 (Fig. 5 C).

Mutating W454A reduced the *G*(*V*) shift for Wu161 (from −9.8 ± 1.1 mV, *n* = 5, to −5.6 ± 0.8 mV, *n* = 4; Fig. 5, E and F; and Table S1), suggesting that Wu161 does bind to the S4/pore site, but its effect on the WT Shaker K_V_ channel is smaller than that of Wu50 (−25.5 ± 1.4; *n* = 5), which may indicate that binding of this compound to this site is less favorable than that of Wu50, in line with the MD simulation results. Removing R362 did not increase the *G*(*V*) shift (−8.5 ± 0.5, *n* = 3), as it did for Wu50 (−49.6 ± 1.2, *n* = 3; Fig. 5 G and Table S1). This may indicate that while Wu50 fits well between R365 and F416 in the absence of R362 and causes channel opening, Wu161 either does not bind here or, when bound, cannot keep the channel in the open state. In an attempt to further weaken a possible binding of Wu161 to the S4/pore site, we tested the triple mutation R362Q/F416A/W454A. The shift was completely eliminated (Table S1). This mutant was, however, surprisingly sensitive to changes in pH (completely blocked at pH 9) and could therefore not be tested for Wu50.

The R362Q/R365Q double mutant reduced the Wu161-induced *G*(*V*) shift compared with R362Q (from −8.5 ± 0.5, *n* = 3, to −5.2 ± 1.2, *n* = 4; Fig. 5 G and Table S1). Such a reduced effect may indicate that this double mutation abolishes binding of this compound entirely to the S4/pore site. To conclude, the flexible compound Wu161 seems to bind to the S4/pore site, but the binding is less stable than for the more rigid compound Wu50, and the *G*(*V*) shifting effects are thus substantially smaller (Fig. 5 G, blue symbols). In contrast, Wu161 bound to the channel with the 2R motif has a large *G*(*V*)-shifting effect (−32.0 ± 2.7 mV, *n* = 6), indicating stable binding to the S3/S4 site when the 2R motif is present (Fig. 5 G, red symbol; and Table S1).

### A possible binding site at the top of the VSD bundle

**Figure 6.**
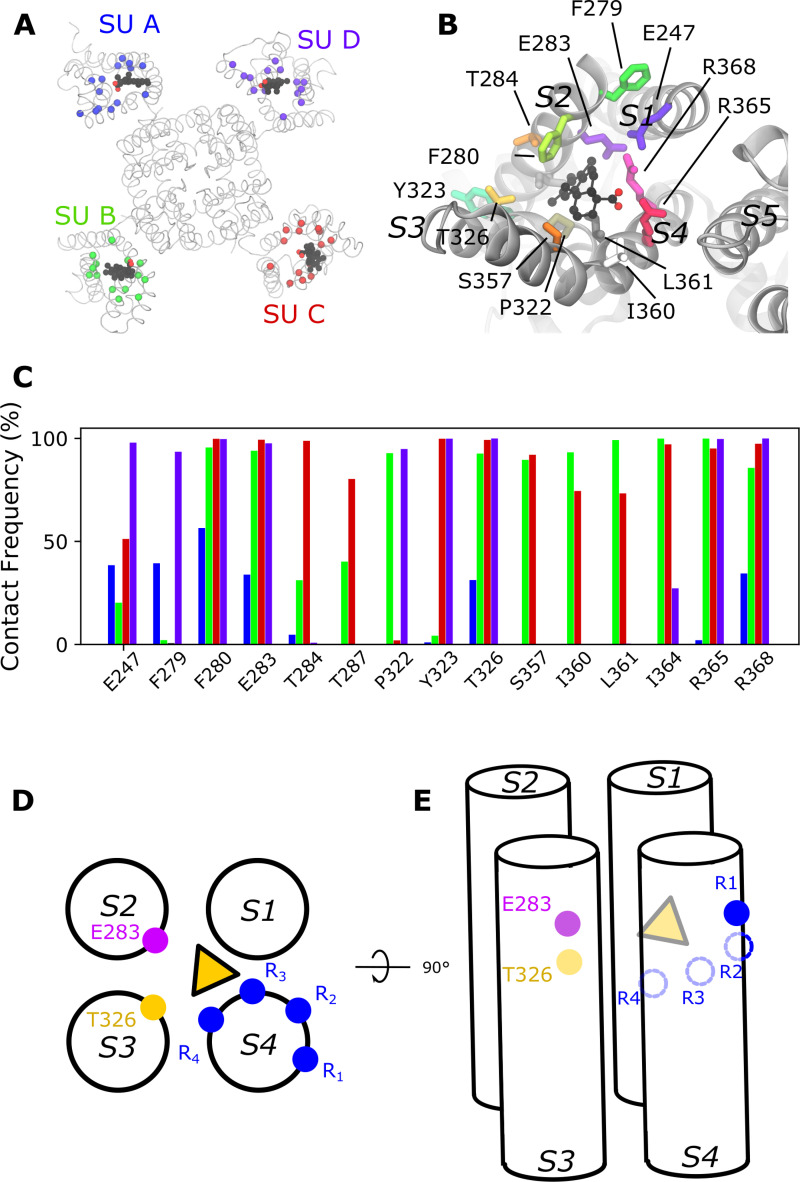
**A possible binding site at the extracellular side of the VSD.** **(A)** Top view of a representative snapshot of the interaction between Wu50 and the top-VSD site in the WT Shaker K_V_ channel. The channel is shown as gray ribbons, the Wu50 compounds are displayed as space filling, each atom type colored differently (black: C or Cl; red: O). Residues are represented as sticks. Residues’ C_α_ coming in contact with Wu50 in at least one of the channel’s subunits (SUs) during MD simulations are represented as spheres and colored according to the subunit they belong to. **(B)** Zoomed-in top view of the top-VSD binding site for the WT Shaker K_V_ channel in the presence of resin-acid derivative Wu50. **(C)** Contact frequency between any heavy atom of Wu50 and the top-VSD site residues in the WT Shaker K_V_ channel simulation. For each residue, each bar corresponds to the contact frequency for one of the four subunits, depicted in a different color, following the color scheme presented in Fig. 1. **(D and E)** Top (D) and side (E) schematic views of the putative effect of resin-acid–derivative binding to the top-VSD site (yellow triangle) The endogenous gating charge arginines (R362 [=R_1_], R365 [=R_2_], R368 [=R_3_], and R372 [=R_4_]) are denoted by blue filled or empty circles. The positions of E283 and T326 are denoted by pink and yellow circles, respectively.

Scrutinizing further the surroundings of S4, we considered a third putative binding pocket in the center of the extracellular region of the VSD (which we call the top-VSD site; Fig. 6). Indeed, positive charges from S4 (R365, R368, and even R371 = R4) are exposed to the extracellular solution, and this site has been shown to be druggable for other classes of compounds (Liin et al., 2018a) and in related channels (Ahuja et al., 2015; Li et al., 2013; Peretz et al., 2010). We thus docked Wu50 to this putative site surrounded by the S1, S2, S3, and S4 helices. Binding there in the O state appears stable in MD simulations (Fig. 6 C), the negative charge of Wu50 being close to R365 and R368. Other residues often in contact with the compound include F280 and E283 in S2 and T326 in S3 (Fig. 6). Experimental tests of the role of these residues via mutagenesis proved difficult; the mutations only showed very small currents or the compound blocked the current, thus precluding studies of the *G*(*V*)-shifting effect (see Table S1).

## Discussion

**Figure 7.**
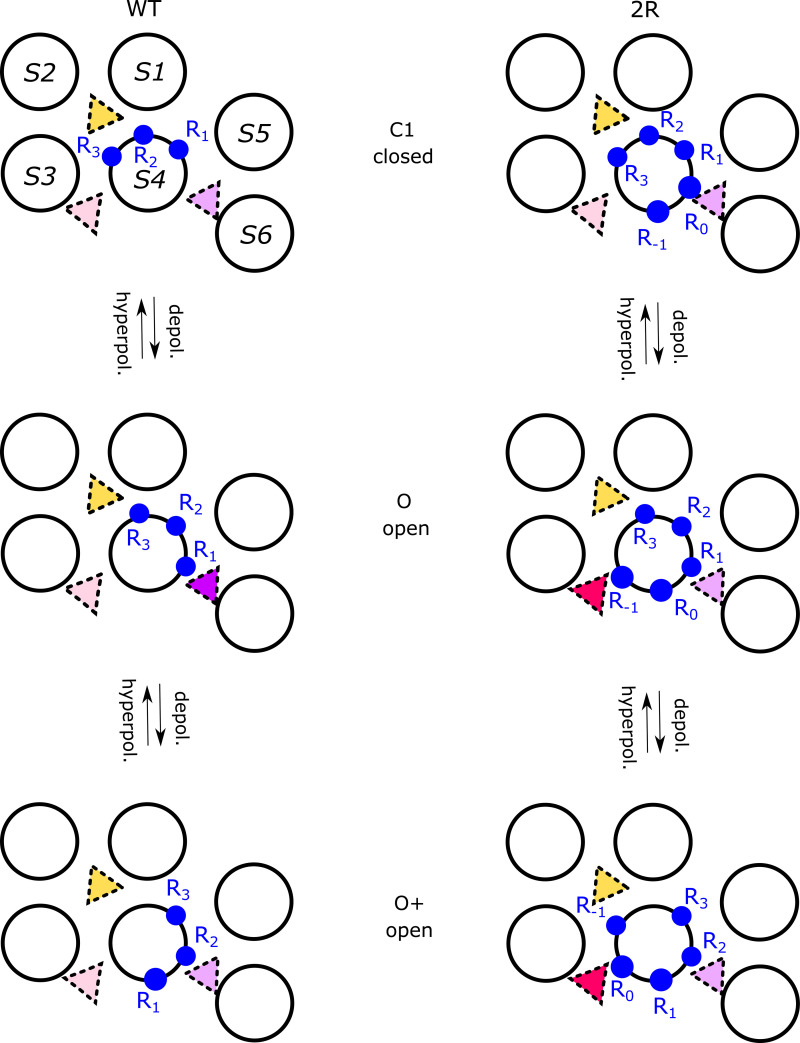
**Three putative resin acid–binding sites act on activation via differential stabilization of S4 in various activation states.** Top views of the motion of S4 during the last steps of activation occurring in one subunit (N.B. the pore domain and VSD are from two different subunits) in the WT Shaker K_V_ channel (left) and the 2R mutant (right), from the C1 closed state (top row) to the open O state (middle row) to the putative open O^+^ state (bottom row). The arrows show the transitions occurring upon depolarization (indicated by depol.) and upon hyperpolarization (indicated by hyperpol.). Binding to the S3/S4 site is represented as a red triangle, to the S4/pore site as a purple triangle, and to the top-VSD site as a yellow triangle. The charged residues are shown as blue filled circles. In the last intermediate (C1) closed state, both for the WT and the 2R channel, binding to the S3/S4 site is weak (light red triangle), binding to the S4/pore site is weak (light purple triangle), and binding to the top-VSD site is strong (yellow triangle). In the open O state, binding to the S3/S4 site is weak (light red triangle) in the WT but strong (bright red triangle) in 2R due to the proximity of R_−1_, binding to the S4/pore site is strong (bright purple triangle) in the WT due to the proximity of R_1_ but presumably weak in the 2R (light purple triangle), and binding to the top-VSD site is strong (yellow triangle) in both the WT and the 2R mutants. In the hyperactivated/open O^+^ state, binding to the S3/S4 site is weak (light red triangle) in the WT but presumably strong (bright red triangle) in 2R due to the proximity of R_0_, binding to the S4/pore site is weak (light purple triangle) in both the WT and the 2R mutants, and binding to the top-VSD site is strong (yellow triangle) in both the WT and the 2R mutants.

The helical-screw motion of S4 combined with the periodic arrangement of arginines every helical turn defines three potential binding sites for resin acids and their derivatives, which can be more or less occupied depending on state- and compound-dependent available interaction motifs (Fig. 7).

### Three tentative binding sites—a summary

Binding of resin-acid compounds to the S3/S4 site (Fig. 7, red triangle) tends to favor S4 activation (Fig. 7, displacement downward from the C1 to the O state) through an electrostatic effect. Indeed, this site is particularly occupied in the activated O state when the 2R motif is introduced, as evidenced by MD simulations and thanks to interactions between the compound and R_−1_. It would also be favorably occupied in a hypothetical hyperactivated O^+^-state channel thanks to the recruitment of R_0_ to this site, although whether the affinity for this site in this state is high or medium remains to be investigated. Binding to this site in the absence of the 2R motif in any state, on the other hand, is seemingly relatively unstable: in the activated O state, R362 (R_1_) and R365 (R_2_) are located on the opposite side of the S4 helix, and R368 (R_3_) and R371 (R_4_) are located too far down, a situation not drastically modified by a transition to the C1 or the O^+^ state.

We further hypothesize that binding to the S4/pore site (Fig. 7, purple triangle) tends to favor S4 activation (transition from C1 to O state) through direct binding to R_1_. The aromatic ringlike body of the resin-acid derivative anchors to W454 or, in its absence, to F416 (Fig. 3, C, D, and I). Removing R_1_ can enhance the effect of binding to this site since its absence presumably favors a transition to an even more activated state O^+^ where R_2_, which is usually buried farther into the S4/S5 crevice, is further pulled upward. What happens in this site in the presence of the 2R motif is not as clear since the action through the S3/S4 site tends to dominate and obscure events at this site.

Finally, binding to the top-VSD site presumably has complex effects (Fig. 7, yellow triangle), with interactions between the resin-acid derivative and R_2_ possibly stabilizing the C1 state, with R_3_ possibly stabilizing the O state and R_4_ possibly stabilizing the O^+^ state. Thus, its effect on the relative stabilization of the different states remains unclear.

### Quantitative evaluation of site-dependent contributions to the *G*(*V*) shifts

In an attempt to quantitatively evaluate the contributions of Wu50 and Wu161 binding to the *G*(*V*) shifts, we fit a simple model assuming that binding to the various sites is independent and that the contributions of compound binding to the *G*(*V*) shift are thus additive (Eq. 4 and Supplemental text). The model further assumed that there is a residual effect that can be due to binding to the VSD-top site or to any other unidentified site and that the contributions due to binding to the S3/S4 site and the S4/pore site depend upon mutations at this site.

The three contributions related to compound binding to each site, ΔV_S3/S4_, ΔV_S4/pore_, and ΔV_Residual_, were estimated using a least squares fit of datasets consisting of *G*(*V*) shifts (ΔV) for Wu50 and Wu161 (Materials and methods and Table S1). The different contributions can be summarized as follows (Fig. S10 A): The residual ΔV_Residual_ is −10 mV for Wu50 (five mutants, constrained to be the same as for eight mutants; see Supplemental text), while it is 0 to −6 mV for Wu161. As mentioned above, the most likely binding site responsible for this shift is the top-VSD site. Our model thus suggests this may not be a binding site for Wu161 or that binding of this compound to this site has no effect on the overall *G*(*V*) shift. Binding to the S3/S4 site in the presence of the 2R mutations contributes with a ΔV_S3/S4_ shift of −17 mV (eight mutants) or −10 mV (five mutants) for Wu50 and with −17 mV (five mutants) for Wu161. This suggests that Wu161 may have a larger effect than Wu50 when binding to this site when the 2R motif is present. The WT S4/pore site contributes with a −15 mV (eight mutants) to −18 mV (five mutants) ΔV_S4/pore_, through binding between W454 and R362 in the O state. Wu161 contributes with −7 to −13 mV. This suggests that Wu50 has a larger effect than Wu161. In the R362Q configuration of the S4/pore site, Wu50 is suggested to bind between W454 and R365 in a hypothetical O^+^ state. The contribution is very large for Wu50, with a ΔV_S4/pore_ of −34 mV (eight mutants) to −37 mV (five mutants). For Wu161 the contribution is only 0 to −6 mV. In the W454A configuration of the S4/pore site, the compounds are suggested to bind between R362 and F416 in the C1 state or R365 and F416 in the O state. Accordingly, the contributions of Wu50 or Wu161 are small (+2 to −6 mV; with no clear difference between the five-mutants and eight-mutants datasets). In the R362Q/W454A configuration of the S4/pore site, the data only come from the eight-mutant model of Wu50. The compound is suggested to bind between F416 and R365 in the O state, and the resulting contribution is large, −31 mV.

We calculated the shift predicted from the model and correlated it with the experimentally measured shift. The correlation obtained was good (Fig. S10 B), supporting the assumption of independent binding sites. It should be noted that the eight-mutant calculation for Wu50 showed the largest deviations for W454A and 2R/W454A, suggesting a putative interaction between these binding sites, as suggested previously.

### The S3/S4 site

Both experimental data and MD simulations have previously been used to describe an interaction site for resin acids in the cleft between S3 and S4 (Fig. 7, red; Ottosson et al., 2017). We also described that the 2R motif (M356R/A359R; Fig. 7, R_−1_, R_0_) greatly enhances the efficacy of resin acids (Ottosson et al., 2014, 2015, 2017; Silverå Ejneby et al., 2018). Here, we have shown that the 2R motif increases the Wu50-induced *G*(*V*) shift by 4–32 mV in the negative direction along the voltage axis, the magnitude of the shift depending on the background mutations. Binding to the S3/S4 site appears to be sensitive to the presence of charges at the extracellular end of S3 and S4. Some channels, such as K_V_2.1, have a native arginine at R_0_ (Fig. S11). For Na_V_ and Ca_V_ channels (which are modulated by the resin acid isopimaric acid; Salari et al., 2018), the charge profile varies from subunit to subunit. By designing resin-acid derivatives that primarily bind to the S3/S4 site, as appears to be the case for Wu161 and Wu181, it might be possible to engineer selectivity for specific channels or specific domains in heteromeric channels. As an example, resin-acid derivatives with a longer and flexible stalk are effective openers of the Shaker K_V_ channel with the 2R motif (Silverå Ejneby et al., 2018; present paper) and the K_V_7.2/7.3 channel (Silverå Ejneby et al., 2018). Thus, modifications of the stalk can be envisioned to obtain compounds selective for different K_V_ channel types.

### The S4/pore site

For the WT Shaker K_V_ channel, the S4/pore site, located between the VSD and the pore (Fig. 7, purple), was found to be occupied by resin-acid derivatives. Binding to this site could possibly be disrupted by mutating W454 to alanine and/or by removing both R362 and R365 (Fig. 3) and was not as favorable for resin acids with a longer, flexible stalk. The presence of aromatic residues (W454 and/or F416 in Shaker) appeared important. A tryptophan in position 454 is unique for the Shaker K_V_ channel among all K_V_ channels (Fig. S11), but K_V_3 and K_V_7 channels have a tryptophan in an adjacent position. F416, on the other hand, is either a phenylalanine or a tyrosine in most K_V_ channels and could potentially contribute to the general binding of resin-acid derivatives. Considering the presence of aromatic residues in various channels may be a way to engineer specificity for various channels (Fig. S11).

### A promiscuous top-VSD site

Removing the 2R motif and disrupting the S4/pore site was, however, not enough to render the channel completely insensitive to Wu50. This residual *G*(*V*) shift could possibly depend on Wu50 binding to another site. Here, we have suggested that Wu50 can bind in a fairly promiscuous drug pocket located in a cleft in the top of the VSD in different voltage-gated ion channels (Fig. 7, yellow; Ahuja et al., 2015; Li et al., 2013; Liin et al., 2018a; Peretz et al., 2010; Li et al., 2020). From here the negatively charged Wu50 could contribute to the negative *G*(*V*) shift by electrostatically attracting S4 charges to rotate S4 in the clockwise direction and favor activation.

### Advantages and challenges of multi-site drug action

Traditional structure-based drug design aims to optimize compounds that fit in and strongly bind to a well-defined pocket of a biomolecule. This work, together with recent developments (Heusser et al., 2018), shows that membrane proteins may be druggable via binding of compounds in a state-dependent manner, in different binding sites with similar binding affinity, and via mechanisms involving the membrane (Ahuja et al., 2015; Lambert et al., 2003; Wang, 2011). The resin-acid derivatives studied in this work seem to possess all of these three properties. These compounds seem to differentially stabilize the activated/open and the intermediate/closed states of the Shaker K_V_ channel depending on their binding to one or more of these three sites. Two of these sites are membrane facing, and binding of the compound to these sites involves displacing a previously bound lipid. While the lipid bilayer has so far often been considered an inert scaffold for membrane proteins, it is now recognized to often play an integral part in modulating protein function and in regulating the access of drug to proteins through competition with the compounds. This indicates that the membrane composition, known to vary from one cell type to the next, also offers possibilities for designing selective drugs with reduced off-target effects. For this to become a rational process, much of the molecular details underlying drug binding remain to be understood. We propose that studying the effects of resin-acid derivatives on a model channel constitutes a first step in this direction. The experimental resolution of other channel states (intermediate and closed) will strengthen this type of work by enabling the explicit modeling and comparison of interactions of drugs with these other states.

Nevertheless, many fascinating questions remain after this work. For example, we were not able to assess binding affinity of the compounds to the different sites, such that we still do not know if the drugs bind in one or more sites at a time, or even if they bind to the same site in all four subunits. Finally, future research directions also involve deciphering the binding mechanism of the drugs to their site, particularly when it involves displacing lipids.

## Supplementary Material

Table S1 Summary of *G*(*V*) shifts and *G*_MAX_ effects induced by 100 μM of either Wu50 or Wu161 for the mutants reported in this paper
