# Supplementary material for: Resin-acid derivatives bind to multiple sites on the voltage-sensor domain of the Shaker potassium channel
Source: J Gen Physiol. 2021 Mar 8;153(4):e202012676. doi: 10.1085/jgp.202012676 (PMC7944402; doi:10.1085/jgp.202012676)
Supplement: Table S1 — Summary of G(V) shifts and GMAX effects induced by 100 μM of either Wu50 or Wu161 for the mutants reported in this paper [file JGP_202012676_TableS1.docx]

**SUPPLEMENTARY TABLE**

**Table S1: Summary of *G*(*V*) shifts and *G*_MAX_ effects induced by 100 μM of either Wu50 or Wu161 for the mutants reported in this paper**

|  | Wu50  pH 9.0 |  |  |  |  |  | Wu161  pH 7.4 |  |  |  |  |  |
| --- | --- | --- | --- | --- | --- | --- | --- | --- | --- | --- | --- | --- |
| **Mutant** | **ΔV_G(V)_ (mV)** |  |  | **G_MAX_ (rel.)** |  |  | **ΔV_G(V)_ (mV)** |  |  | **G_max_ (rel.)** |  |  |
|  | Mean | SEM | n | **Mean** | SEM | n | Mean | SEM | n | **Mean** | SEM | n |
|  |  |  |  |  |  |  |  |  |  |  |  |  |
| **M356R/A359R (=2R)** | -40.0 | 2.7 | 10 | 1.58 | 0.09 | 8 | -32.0 | 2.7 | 6 | 1.20 | 0.05 | 6 |
| **M356R/A359R/W454A** | -39.6 | 2.9 | 5 | 1.28 | 0.09 | 5 |  |  |  |  |  |  |
| **M356R/A359R/R362Q** | -54.0 | 2.3 | 4 | 1.93 | 0.25 | 4 | -19.6 | 4.1 | 5 | 1.19 | 0.07 | 5 |
| **M356R/A359R/R362Q/W454A** | -56.6 | 5.8 | 4 | 1.50 | 0.14 | 4 |  |  |  |  |  |  |
|  |  |  |  |  |  |  |  |  |  |  |  |  |
| **WT** | -25.5 | 1.4 | 5 | 1.36 | 0.07 | 6 | -9.8 | 1.1 | 5 | 1.03 | 0.04 | 3 |
| **W454A** | -8.1 | 0.4 | 5 | 0.56 | 0.08 | 5 | -5.6 | 0.8 | 4 | 0.86 | 0.04 | 4 |
| **R362Q** | -49.6 | 1.2 | 3 | 1.25 | 0.14 | 3 | -8.5 | 0.5 | 3 | 1.05 | 0.01 | 4 |
| **R362Q/W454A** | -41.0 | 4.4 | 5 | 1.28 | 0.08 | 5 |  |  |  |  |  |  |
| **R362Q/W454A/F416A** | -34.3 | 3.4 | 3 | 0.17 | 0.06 | 5 | 2.0 | 1.4 | 4 | 0.47 | 0.07 | 4 |
|  |  |  |  |  |  |  |  |  |  |  |  |  |
| **R362Q/R365Q** | -25.5 | 4.6 | 5 | 0.55 | 0.12 | 5 | -5.2 | 1.2 | 4 | 0.99 | 0.03 | 4 |
| **R362Q/R365Q/W454A** | -24.7 | 3.0 | 5 | 0.57 | 0.13 | 6 |  |  |  |  |  |  |
| **R362Q/R365Q/W454A/F280I** | ND | N/A | N/A | 0.05 | 0.02 | 4 | 1.1 | 3.1 | 3 | 0.64 | 0.12 | 3 |
